# Molecular characterization, receptor binding property, and replication in chickens and mice of H9N2 avian influenza viruses isolated from chickens, peafowls, and wild birds in eastern China

**DOI:** 10.1080/22221751.2021.1999778

**Published:** 2021-11-12

**Authors:** Jing Guo, Yanwen Wang, Conghui Zhao, Xinxin Gao, Yaping Zhang, Jiqing Li, Mengjing Wang, Hong Zhang, Wenqiang Liu, Chao Wang, Yingju Xia, Lu Xu, Guimei He, Jinyan Shen, Xiaohong Sun, Wenting Wang, Xinyu Han, Xiaoxuan Zhang, Zhengyang Hou, Xinlin Jin, Na Peng, Yubao Li, Guohua Deng, Pengfei Cui, Qianyi Zhang, Xuyong Li, Hualan Chen

**Affiliations:** a College of Agronomy, Liaocheng University, Liaocheng, People’s Republic of China; b State Key Laboratory of Veterinary Biotechnology, Harbin Veterinary Research Institute, Chinese Academy of Agricultural Sciences, Harbin, People’s Republic of China; c National Classical Swine Fever Reference Laboratory, China Institute of Veterinary Drug Control, Beijing, People’s Republic of China; d Institute of Eco-Chongming (IEC), East China Normal University, Shanghai, People’s Republic of China

**Keywords:** Avian influenza virus, H9N2, chicken, peafowl, wild birds

## Abstract

H9N2 avian influenza viruses are widely prevalent in birds and pose an increasing threat to humans because of their enhanced virulence and transmissibility in mammals. Active surveillance on the prevalence and evolution of H9N2 viruses in different avian hosts will help develop eradication measures. We isolated 16 H9N2 viruses from chickens, green peafowls, and wild birds in eastern China from 2017 to 2019 and characterized their comparative genetic evolution, receptor-binding specificity, antigenic diversity, replication, and transmission in chickens and mice. The phylogenetic analysis indicated that the green peafowl viruses and swan reassortant shared the same ancestor with the poultry H9N2 viruses prevalent in eastern China, while the seven wild bird viruses belonged to wild bird lineage. The chicken, peafowl, and swan H9N2 viruses that belonged to the poultry lineage preferentially recognized α-2, 6-linked sialic acids (human-like receptor), but the wild bird lineage viruses can bind both α-2, 3 (avian-like receptor) and human-like receptor similarly. Interestingly, the H9N2 viruses of poultry lineage replicated well and transmitted efficiently, but the viruses of wild bird lineage replicated and transmitted with low efficiency. Importantly, the H9N2 viruses of poultry lineage replicated in higher titer in mammal cells and mice than the viruses of wild birds lineage. Altogether, our study indicates that co-circulation of the H9N2 viruses in poultry, wild birds, and ornamental birds increased their cross-transmission risk in different birds because of their widespread dissemination.

## Introduction

Influenza A viruses are negative-sense RNA viruses, which comprise eight gene segments, including surface genes hemagglutinin (HA) and neuraminidase (NA), and internal gene backbone, basic polymerase 2 (PB2), basic polymerase 1 (PB1), acidic polymerase (PA), nucleoprotein (NP), matrix (M), and nonstructural protein (NS) genes. Influenza A viruses are categorized into 18 different HA and 11 different NA subtypes based on antigenic differences in their surface glycoproteins. Except for the H17N10 and H18N11 influenza A viruses detected from bats, 16 HA and 9 NA virus subtypes have been isolated from avians [1, 2]. Nearly all of the avian influenza viruses (AIVs) reside in their natural hosts: wild birds, and only a few subtypes, such as H5N1, H5N6, H7N9, and H9N2 viruses, have been successfully transmitted to poultry and have become epidemic in poultry [3–7].

H9N2 AIVs were first isolated in turkeys in 1966 [8]. Over half a century of worldwide prevalence, H9N2 viruses have become enzootic in poultry in the Eurasian region [9–11]. H9N2 AIVs have been reported to infect a broad range of avian hosts, including chickens, ducks, geese, pigeons, quails, ostriches, wild waterfowls, and wild terrestrial birds [12–16]. Our previous study found that the H9N2 viruses isolated in China from 2009 to 2013 can transmit between ferrets by respiratory droplets, which posed an increasing threat to public health [17, 18]. From 1998 to June 2021, a total of 87 H9N2 human infection cases have been reported in China [19–26]. Especially, the human H9N2 infection case number from 2015 to 2021 was approximately 69% (60 cases) of the total cases (87 cases). Nearly all individuals infected with H9N2 AIVs have been confirmed to have poultry experience exposure, which indicated that the H9N2 viruses could transmit directly from avian species to humans. In addition, H9N2 viruses have been confirmed to contribute to the genesis of novel H7N9 and H5N6 reassortants, which caused poultry and human infections [27, 28]. These findings emphasize that H9N2 influenza viruses have sustained challenges to birds and have the potential risk of causing epidemics in humans.

The wild birds, especially the migratory wild waterfowls, are the natural reservoir of AIVs. The low pathogenic avian influenza viruses (LPAIV) were mainly replicated in the respiratory and digestive tracts of wild birds with mild or subclinical infections. The viruses were then excreted with droppings to contaminate the water and the environment of the habitat. Additionally, the co-existence of the wild birds and the poultry around the wetland or lake located in the important migratory flyway increased cross-transmission of AIVs between wild birds and poultry and thus promoted the emergence of novel reassortants. H9N2 AIVs have been detected in kinds of wild birds, such as anseriforme, falconiformes, and galliformes [16, 29, 30, 31, 32]. Therefore, active surveillance of AIVs in wild birds is helpful for fully understanding the ecology of the viruses and evaluating their transmission risk.

In this study, we isolated two H9N2 AIVs from green peafowls in a park, and eight H9N2 viruses from wild migratory waterfowls in wetlands of eastern China, in 2019. We also selected six H9N2 viruses isolated from chickens in live poultry markets (LBMs) and poultry farms in the neighborhood during 2017 and 2018 to investigate their comparative evolutionary relationships, receptor-binding properties, antigenic diversity, neuraminidase activity, replication, and transmissibility in chickens and mice.

## Materials and methods

### Ethics statements and facility

The animal studies were carried out according to the Guide for the Care and Use of Laboratory Animals of the Ministry of Science and Technology of the People's Republic of China. All experiments with infectious H9N2 viruses were conducted within the animal biosafety level 2 (ABSL-2) facility. The animals used in this study were placed in the biological safety isolator. The researchers who work with mice and chickens wear N95 masks and disposable overalls.

### Samples collection and viruses isolation

The throat and cloacal swabs, tissue samples of the chickens, fresh droppings of the green peafowls, swans, and wild migratory waterfowls were placed into 2 ml of minimal essential medium supplemented with penicillin and streptomycin. The virus was isolated by using 10-day-old embryonated chicken eggs. The hemagglutinin (HA) subtype was determined using the HI test, and the neuraminidase (NA) subtype was determined by PCR and genetic sequencing. The viruses were stocked in a −80°C freezer, and the virus stocks were grown in specific pathogen-free (SPF) chicken eggs.

### Cell lines

Madin-Darby canine kidney (MDCK), Human lung adenocarcinoma epithelial (A549), and 293 T cell lines were purchased from the Cell Resource Center of the Shanghai Institute of Life Sciences and preserved by our laboratory. Chicken embryo fibroblast (CEF) monolayer cells were made from the 10 days old SPF chicken embryonated eggs. MDCK, CEF and 293 T cells were grown in Dulbecco's modified Eagle's medium (DMEM) containing 10% fetal bovine serum (FBS) and antibiotics. A549 cell lines were grown in an F-12 K nutrient mixture containing 10% FBS and antibiotics. All cells were cultured at 37°C with 5% CO2.

### Molecular and phylogenetic analyses

The PCR products of eight segments of five H9N2 viruses were sequenced by the specific sequencing primers (primer sequences available on request). Sequence data were compiled with the SEQMAN programme (DNASTAR, Madison, WI) according to the reference sequences. Phylogenetic analyses were carried out with the PHYLIP programme of MEGA 7.0 software using the neighbor-joining algorithm. Bootstra*p* values of 1000 were used.

### Bayesian Phylogenetic analysis

Bayesian time-measured phylogenetic trees of HA and NA gene segments of H9N2 viruses were constructed using the BEAST software package (v1.10.4) [33] under the SRD06 codon partition and the general time-reversible (GTR) nucleotide substitution model. Both analyses were performed with a relaxed uncorrelated lognormal molecular clock model, and the constant size coalescent tree prior was selected according to the path sampling and stepping-stone sampling marginal likelihood estimation [34]. Markov Chain Monte Carlo (MCMC) chains were run for 50 million generations, sampling every 5000 steps. Tracer (v1.7.1) [35] was applied to check the parameters of trees for an effective sample size (ESS) larger than 200. The initial 10% states were disregarded as the burn-in, then the maximum clade credibility (MCC) trees were reconducted by TreeAnnotator of the BEAST package and then visualized by the FigTree software (v1.4.4).

### Receptor property analysis

The methods of receptor-binding analysis using a solid-phase direct binding assay have been described previously [17, 36]. Our laboratory synthesized two different glycopolymers: α-2, 3-siaylglycopolymer [Neu5Aca2-3Galb1-4GlcNAcb1-pAP (para-aminophenyl)-alpha-polyglutamic acid (α-PGA)] and α-2, 6-sialylglycopolymer [Neu5Aca2-6Galb1-4GlcNAcb1-pAP (para-aminophenyl)-alpha-polyglutamic acid (α-PGA)], to test the receptor-binding specificity of the H9N2 viruses [37, 38]. Dose–response curves of virus binding to the glycopolymers were analyzed using a single-site binding algorithm and curve-fitting by GraphPad Prism to determine the associated constant values (Ka). Each value is presented as the mean ± SD of three experiments, which were each performed in triplicate.

### Antigenic analysis

The chicken antisera used in this study were generated in SPF chickens that were inoculated with 10^6^EID_50_ in a volume of 200 µl. Antigenic analysis was performed by using the HI assay with 1% chicken erythrocyte. The antigenic map of the H9N2 viruses used in this study was generated using the HI assay data in Origin software.

### Neuraminidase assay

The neuraminidase activity was performed in the presence of the substrate 2′-(4-methylumbelliferyl)-α-d-N-acetylneuraminic acid (MUNANA) as described previously [39]. Briefly, the viruses were serially 2-fold diluted from 1×10^7^ EID_50_/ml to 9.8×10^3^ EID_50_/ml. The copy number of the M gene was quantitated by relative real-time PCR to ensure accurate accuracy of diluted virus dose [40]. Then, 50 μl of 200 μM MUNANA was mixed with 50 μl of virus dilution and incubated at 37°C for 60 min, and the reaction was stopped by adding 100 μl of 0.2 M Na_2_CO_3_. At last, The fluorescence was measured at excitation and emission wavelengths of 365 nm and 450 nm, respectively. The Neuraminidase activity was performed in triplicate. The luciferase activities were measured on an Enspire Multimode Plate Reader (PerkinElmer).

### Luciferase assay of polymerase activity

A dual-luciferase reporter assay system was performed as described previously [41]. Briefly, the reporter plasmids consist of the firefly luciferase open reading frame and flanked by non-coding regions of NP of influenza A viruses, under human RNA polymerase I promoter and terminator control. The reporter plasmid Ppol I -LUC-NP, together with the pTK-RL (Promega) and pCAGGs plasmid constructs expressing the polymerase PB2, PB1, PA, and NP genes (100 ng each) plus Lipofectamine LTX (Invitrogen) were transfected into 293 T cells as recommended by the manufacturer's protocol. pTK-RL is an internal control plasmid to normalize transfection efficiency that encodes the Renilla luciferase protein. Cell extracts were harvested 48 hpi, and luciferase activity was detected using the luciferase assay system (Promega). The assay was standardized against the Renilla luciferase activity. All experiments were performed in triplicate.

### Viral growth curves in cells

Viruses were diluted to 10^6^ EID_50_/0.2 ml and were inoculated into MDCK, A549, and CEF monolayers in a volume of 200 µl/well. One hpi, the cells were replaced with fresh OPTI-MEM and incubated at 37°C. Virus-containing culture supernatant was collected at various time points and titrated in eggs. The growth data shown are the average results of three independent experiments.

### Chicken experiments

Five groups of three 6-week-old specific-pathogen-free (SPF) chickens housed in isolator cages (150 cm × 85 cm × 100 cm) were inoculated i.n. with 10^6^ EID_50_ of each virus in a volume of 200 µl to evaluate the viral replication in chickens. On day 3 p.i., the birds in each group were euthanized, and the trachea, lung, spleen, liver, kidneys, pancreas, brain, and intestine were collected for virus titration in eggs.

For the transmission experiment, three chickens were inoculated with the test viruses, and then another three chickens were housed in the same isolator after 24 h p.i. for testing the transmissibility of the viruses in chickens. Tracheal and cloacal swabs of the chickens were collected on days 1, 3, 5, 7, 9, and 11 p.i., respectively, and titrated in eggs. The serum of the chickens was collected on days 10, 15, and 21 p.i. and the antibody titer was detected by the HI test. The chickens were then euthanized on day 21 p.i..

### Mouse experiments

Groups of six-week-old female BALB/c mice (Ji'nan Pengyue experimental Animal Breeding CO., Ltd., Shandong, China) were anesthetized with CO_2_ and inoculated intranasally (i.n.) with 10^6^ EID_50_ of test viruses in a volume of 50 µl. Three mice were euthanized on days 3 and 5 p.i., respectively, and the nasal turbinate, lung, kidneys, spleen, and brain were collected for virus titration in eggs. Three mice were euthanized on days 3 and 5 p.i., respectively, and the lungs were sampled for histologic study. The lung samples were collected, fixed in 10% formalin, and then stained with hematoxylin and eosin (HE). The remaining five mice in each group were monitored daily for 14 days for weight loss and survival.

### Statistical analysis

Quantitative data are presented as means ± SD of at least three biological replicates. Data were statistically analyzed with a two-tailed unpaired Student's *t*-test or a one-way ANOVA followed by a *t*-test using GraphPad Prism 8.0 software. Statistical parameters are reported in the figures and figure legends. *P* values < 0.05 were considered statistically significant.

## Results

### Sampling overview and molecular characteristics of H9N2 AIVs

From 2017 to 2019, a total of 2816 samples were collected from a poultry farm, LBMs, wildlife park, and wild waterfowls habitats in Shandong province, eastern China and total 45 H3, H4, H6, and H9 isolates were identified (Table S1). We collected 73 fresh dropping samples of healthy green peafowl in a park (total peafowl number is 153) once a time on February 22, 2019, and two H9N2 viruses were isolated from the samples (Table S1). 574 fresh droppings of swans were collected in the swan lake wetland of eastern China in 2019, and one H9N2 virus was successfully isolated. A total of 2039 droppings of wild migratory waterfowl were sampled in migratory wild birds habitats in the Yellow River Delta in 2019, and seven H9N2 viruses were isolated (Table S1). The chicken virus, CK/863/17, was isolated from the 15 dead chickens in a poultry farm in 2017. The other five chicken viruses used in this study were isolated from the throat and cloacal swabs of chickens in LBMs from 2017 to 2018 in eastern China (Table S1).

To identify the comparative genesis of the H9N2 viruses, we chose seven wild birds viruses, one swan virus, two peafowl viruses, and six chicken viruses to study their genetic relationships (Table S1). Several significant amino acids differences were observed between different viruses. The amino acid motif at the cleavage site of the HA of the seven wild birds viruses are -ASNR-, while the two green peafowl viruses and one swan virus shared the same sequence motif with the chicken viruses (-KSSR-, -RSSR-, or -RSRR-) (Table S2). The key amino acid substitutions related to the enhanced replication or transmission of AIVs in mammals were compared in the sixteen H9N2 viruses. The amino acid mutation I155 T in HA (H3 numbering) was conserved in 16 isolates [17]. HA-183H, 190E, and 226Q were conserved in the seven wild bird lineage viruses, while the nine viruses that belong to poultry lineage possessed 183N, 190A/V, and 226L mutations in their HA, which contribute to the increased receptor binding to human-like receptors of AIVs (Table S2) [17, 42]. Interestingly, The E627 K mutation in PB2 was not found in all of the viruses; however, PB2-627 V mutation was detected in three chicken isolates demonstrated to enhance the virulence of H7N9 viruses in mice (Table S2). Taken together, these key amino acids variations between the poultry and wild bird viruses implied that the difference in their biological properties.

### Phylogenetic analysis of H9N2 viruses

**Figure 1.**
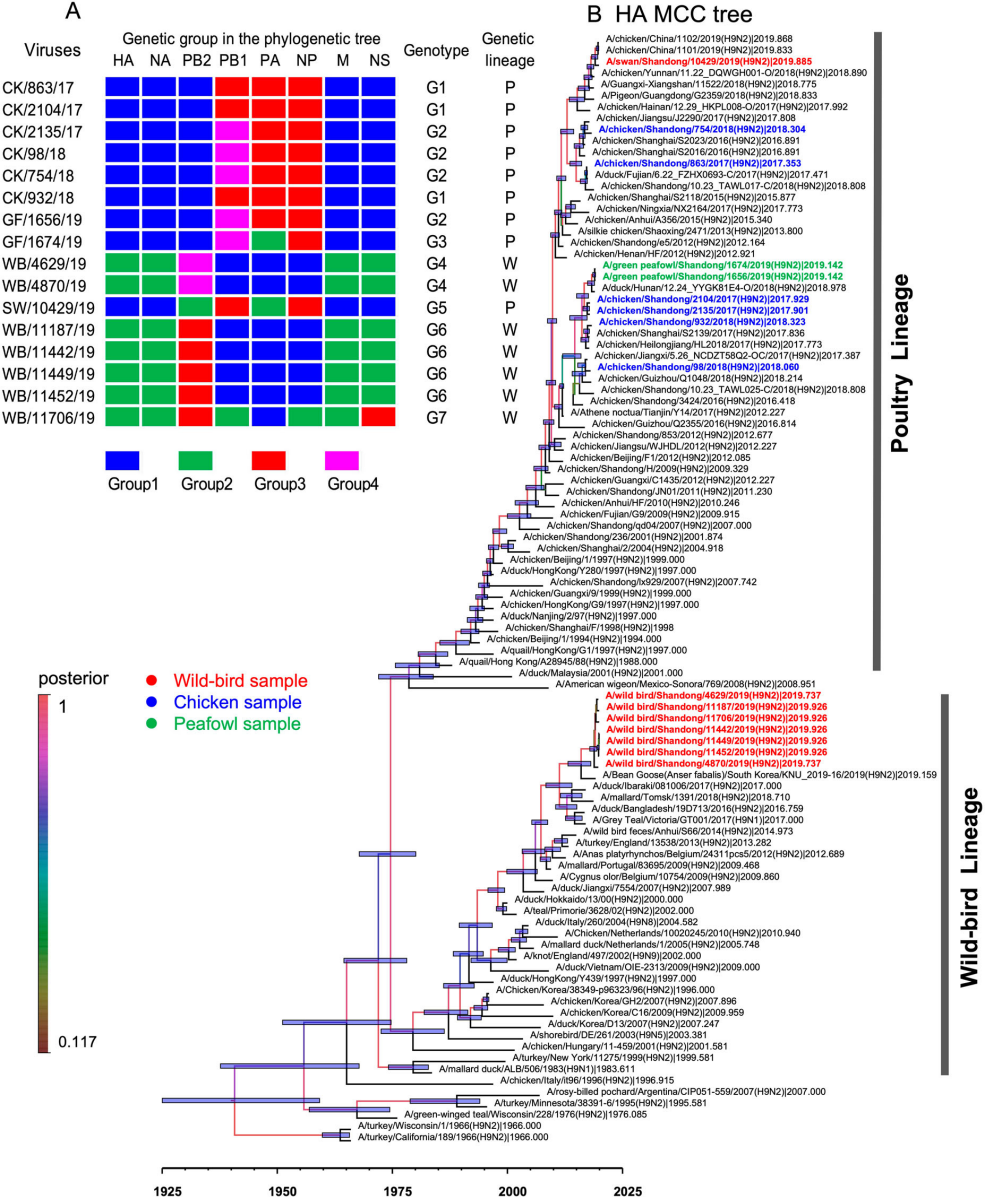
Genetic relationships among the HA gene and genotypes of H9N2 influenza viruses isolated from chickens, peafowls, and wild birds. (A) The genotype of the 16 H9N2 viruses. Groups were divided in each phylogenetic tree as shown in Figure 1B, Figure 2B, Figure 3, and Figure S1 according to their nucleotide homology, then genotypes were confirmed according to the group combination of eight gene segments of each virus. The genetic lineage indicates the genetic lineage (poultry-originated lineage, P; or wild bird originated lineage, W). (B) Bayesian time-measured phylogenetic tree of HA genes of H9N2 viruses. The HA MCC tree was constructed with the BEAST software package (v1.10.4) and then visualized by FigTree (v1.4.4). Branches are coloured by posterior probability, and the node bars indicate 95% highest posterior density of the node height. Tip labels in black are reference sequences downloaded from the GISAID database, and the left 16 sequences in this study are coloured according to their hosts.

**Figure 2.**
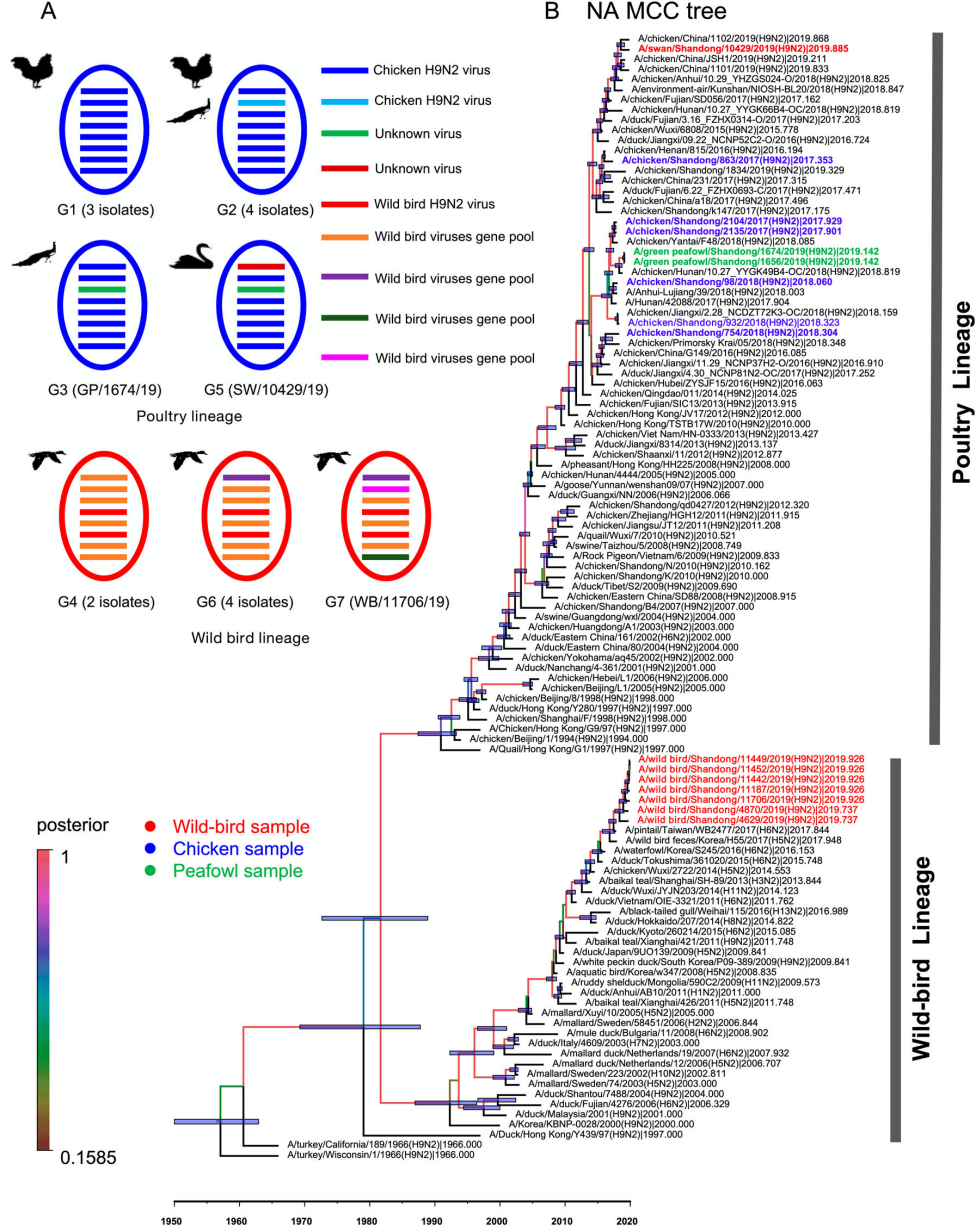
**Genetic relationships among the NA genes and genotype evolution of H9N2 viruses.** (A) Genetic evolution and reassortment of H9N2 viruses. (B) Bayesian time-measured phylogenetic trees of NA genes of H9N2 viruses. The NA MCC tree was constructed with the same software package and method as the Figure 1 legend. The 16 sequences in this study are coloured according to their hosts, as shown in the figure.

To better understand and mapping the evolutionary trend and divergence of HA and NA surface genes of the H9N2 viruses in poultry and wild birds, we constructed the MCC trees of HA and NA by using the Bayesian inference of phylogeny. As shown in the MCC tree of HA, the HA gene was separated into two major branches. The sequences of chicken, peafowl viruses, swan viruses were clustered into the branch of poultry lineage, while the other seven wild bird viruses were clustered into the branch of wild bird lineage (Figure 1(b)). The HA genes of the 16 viruses used in this study shared 81.7-99.9% identity at the nucleotide level, and the nucleotide identity of nine HA genes of poultry lineage was 92.5–99.9% and formed two sub-branches. The seven HA genes of wild bird lineage shared 99.2–99.9% identity at the nucleotide level (Figure 1(b)). The NA gene was separated into two major branches. The NA genes of the 16 H9N2 viruses shared 80.7–100% nucleotide identity. The chicken, green peafowl, and swan viruses were clustered into a branch of poultry lineage and shared 94.3–99.9% nucleotide identity. The NA genes of the seven wild birds viruses shared 98.2–100% identity and clustered into wild bird lineage (Figure 2(b)).

**Figure 3.**
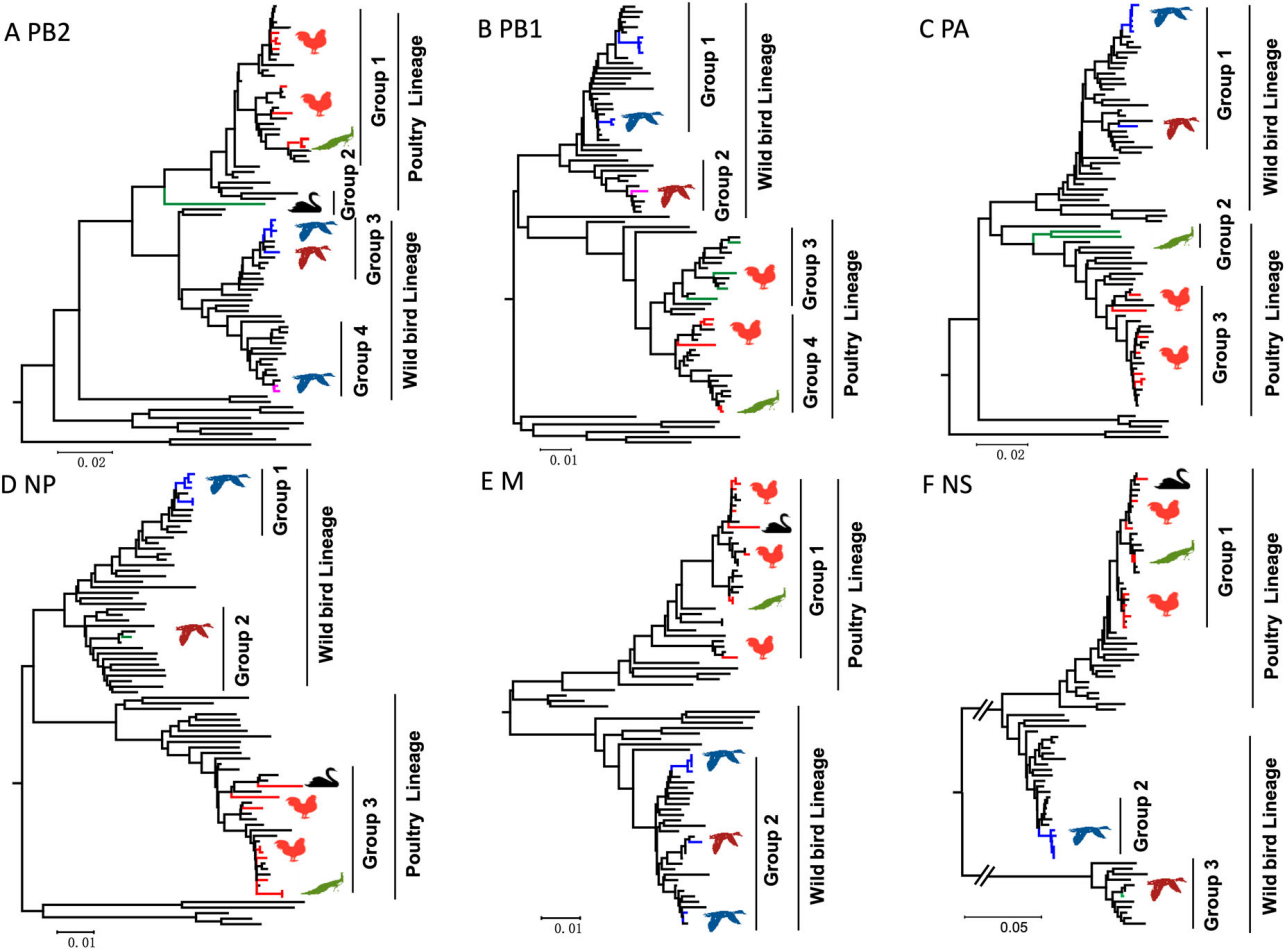
**Phylogenetic diagram of PB2 (A), PB1 (B), PA (C), NP (D), M (E), and NS (F) genes of H9N2 avian influenza viruses.** The phylogenetic tree was constructed by MEGA 7.0 with the Neighbor-Joining method. The virus name was not shown in the tree. The branches and the sequence name with colour (red, blue, green, pink) were the viruses isolated in this study, and the sequences with black were download from the database. Larger versions of the images of the phylogenetic trees, with more detailed sequence information, were provided in Figure S1 A-F in the supplemental part.

The PB2, PB1, PA, NP, M, and NS genes sharing 88.3–100%, 88.3–100%, 88.7–99.9%, 89.2–100%, 89.6–100%, and 69.2–100% identity, respectively. Two major branches, poultry and wild bird lineage were formed in the phylogenetic trees of the six internal genes. The viruses prevalent in wild birds and ducks in the Eurasian region clustered into wild bird lineage, while the viruses that mainly populated in poultry formed the poultry lineage (Figure 3). We then divided the 16 H9N2 viruses into seven genotypes (G1-G7) based on different gene segment group combinations. The viruses in G1, G2, G3, and G5 are clustered into poultry lineage, and the viruses G4, G6, and G7 are clustered into wild bird lineage (Figure 1(a)).

Phylogenetic analysis indicated that the two peafowl viruses’ HA, NA, PB2, PB1, NP, M, and NS genes were highly homologous in the phylogenetic trees, indicating that these genes derived from the same genes ancestor H9N2 virus. However, the PA gene of the two peafowl viruses shared 97.7% identity, which implied that the PA gene might derive from different parent viruses (Figure 2(a)). In addition, we found that the two peafowl H9N2-like viruses as gene donors continued to result in new reassortant H9N2 viruses (Figure 3, Figure S1A-F) (17, 33). Unlike other wild bird viruses used in this study, the HA, NA, PB1, NP, M, and NS genes of the swan virus SW/10429/19 shared the same ancestor with the chicken H9N2 viruses, and the PB2 and PA genes were originated from unknown viruses (Figure 2(a)). Thus, the SW/10429/19 virus was a novel reassortant with donor genes from poultry viruses and unknown viruses. These phylogenetic data indicate that the prevalent H9N2 viruses in nature can lead to cross-transmit between wild and domestic birds by viral reassortment, which in turn broadened their host range and increased cross-species transmission risk.

### H9N2 influenza viruses of poultry lineage preferentially bind to human-like receptors

**Figure 4.**
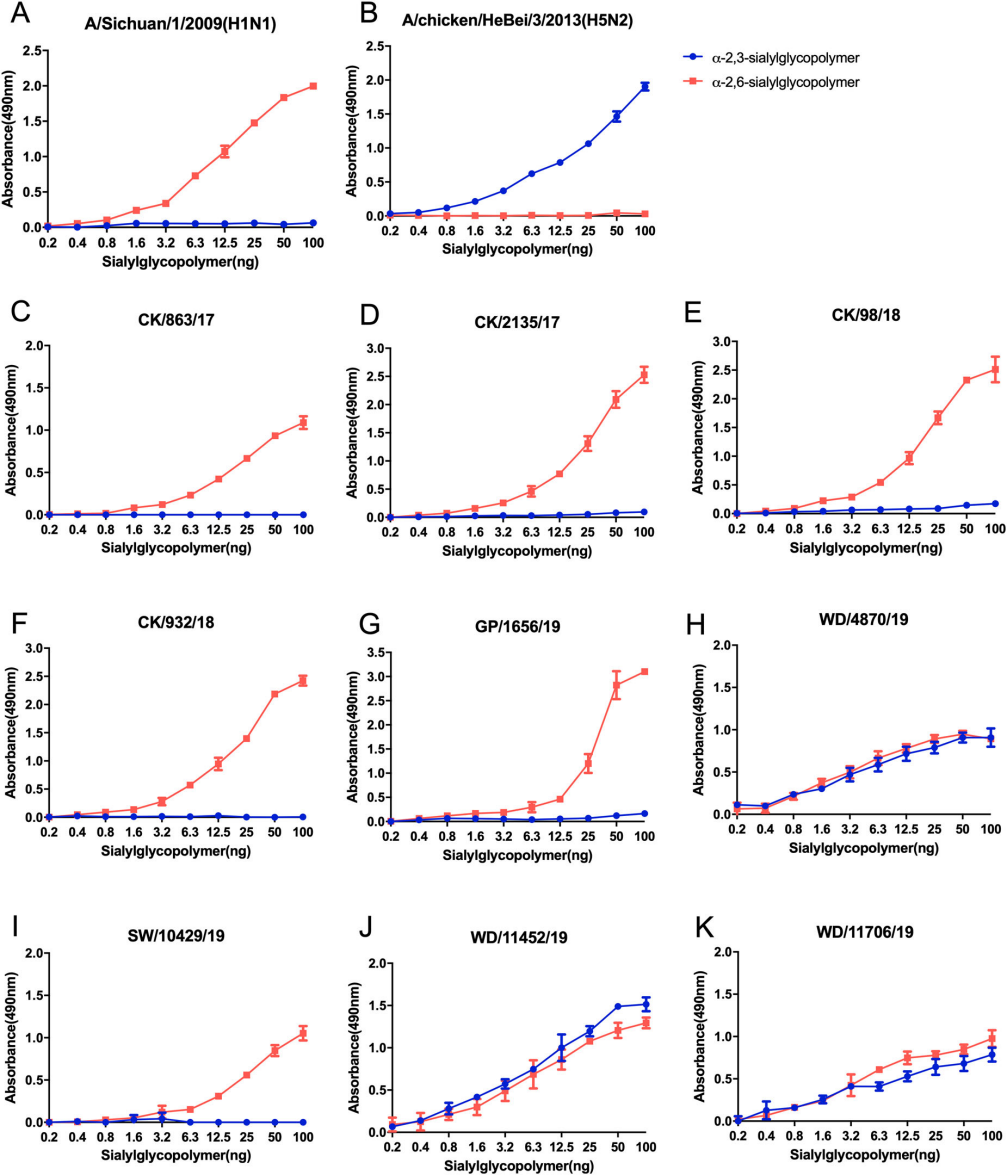
**Characterization of the receptor-binding specificity of H9N2 viruses.** The binding affinity of the test viruses to two different glycopolymers was tested. The data shown are the mean of three replicates; the error bar indicates the standard deviation.

The receptor-binding specificity plays a critical role in the viral replication and transmission between avians and mammals. Here, we used the following two different glycopolymers: the α-2, 3-siaylglycopolymer and the α-2, 6-sialylglycopolymer to test the receptor-binding preference of the H9N2 viruses. The six tested viruses, including four chicken isolates, one green peafowl isolate, and one swan isolate, preferentially bind to α-2,6-linked sialic acids (Figure 4). However, the two H9N2 viruses of wild bird lineage used in this study bind to α-2, 3-linked sialic acids and α-2,6-linked sialic acids in a similarity, which were significantly different from the viruses of the poultry lineage (Figure 4). Our previous study found that the naturally isolated poultry H9N2 viruses from 2009–2013 in China showed a clear preference for α-2,6-linked sialic acids, although they retained their ability to bind to α-2, 3-linked sialic acids, while the poultry H9N2 viruses isolated between 1996 to 2001 can bind both to α-2, 3-linked sialic acids and α-2,6-linked sialic acids with no significant difference [17]. These results indicate that the H9N2 viruses populated in poultry preferentially bind to human-like receptors than those prevalent in wild birds.

### NA activities of the H9N2 viruses

Previous studies showed that the NA proteins promote progeny virions released by cleaving the sialic acids on the host cell surface, contributing to virus replication in host cells [40, 43–45]. Accordingly, we next test the enzymatic activities of NA of the selected nine H9N2 viruses. As shown in Figure S2, the enzymatic activities of CK/863/17, CK/98/18, and SW/10429/19 were similar but higher than the other six viruses (Figure S2). These results indicate that the poultry lineage viruses’ relative high NA activities may further contribute to the replication or transmission of H9N2 viruses.

### Antigenic diversity of the H9N2 AIVs

To fully understand the antigenic diversity of the H9N2 viruses used in this study, we performed an HI assay to detect the HI antibody titer. All the 16 viruses were used here to investigate their cross-reactivity with chicken antisera, and the antigenic analysis data indicated that the cross-reactive HI antibody titer was at a range of 0–1024. The chicken antisera of GP/1656/19 can react with the 16 viruses at range 16–1024, and antisera of WD/4870/19 can react with 16 viruses at range 4–512. The other three antisera reacted poorly with tested viruses (Table S3, Figure S3). These data demonstrate that the H9N2 viruses, especially the poultry lineage virus, are undergoing significant antigenic drift.

### Replication and transmission of H9N2 viruses in chickens

**Figure 5.**
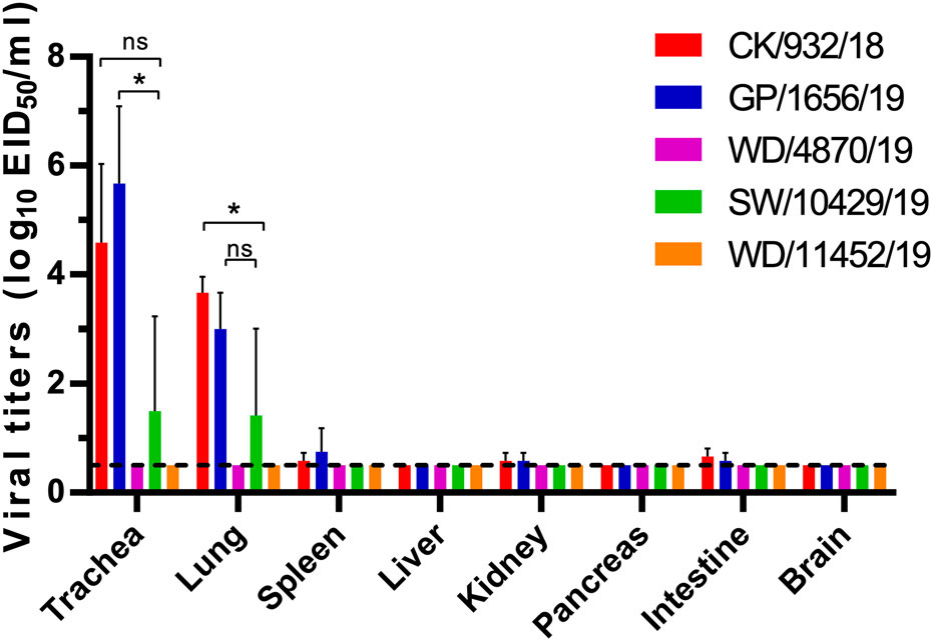
**Replication of H9N2 viruses in chickens.** Viral titers in the organs of chickens on day 3 p.i. with 10^6^EID_50_ of test virus. Data shown are the mean with SD titers from three chickens. The dashed line indicates the lower limit of detection. The virus titers of CK/932/18 and GP/1656/19 in the trachea and lung of chickens were compared with the SW/10429/19. Statistical analysis was performed by using one-way ANOVA with GraphPad Prism 8 software. *, *P* < 0.05; ns, not significant.

We selected five viruses to study the comparative replication and transmissibility of the viruses in chickens (Figure 5, Table 1, Table S4). Interestingly, a significant replication difference of the H9N2 viruses in chickens was observed. The poultry lineage viruses, including chicken, peafowl, and swan viruses, can replicate in the trachea and lung of the chickens, but CK/932/18 and GP/1656/19 replicated more efficiently than SW/10429/19. The viral titers of CK/932/18 and GP/1656/19 in the trachea were higher than the lung, indicating that the two viruses were suitable for replicating in the upper respiratory tract. Low viral titers were detected in the spleen, kidneys, and intestine, and no virus was detected in the liver and pancreas of the chickens infected with CK/932/18 and GP/1656/19. Low viral titers can be detected in the trachea and lung, implying that the unique SW/10429/19 reassortant has limited replication in chickens (Figure 5). Notably, the two viruses, WB/4870/19 and WB/11452/19 were not detected in any organ of the chickens, which suggested the wild bird originated H9N2 viruses have not adapted to replicate in chicken (Figure 5). These results suggested that the H9N2 viruses in poultry replicated well in respiratory organs with limited tissue tropism. However, the wild bird-originated viruses still need specific adaptation before they can replicate efficiently in chickens.

Next, we tested the transmissibility of the five H9N2 viruses in chickens. In the transmission study, the tracheal and cloacal swabs of inoculated and housed chickens were detected in eggs. In the CK/932/18 group, the virus can be detected in the trachea from day 1–7 p.i. in the inoculated chickens, while the virus can be detected in cloacal swabs on day 5 p.i. The CK/932/18 virus can successfully transmit to the housed three chickens, and viruses were detected in the tracheal and cloacal swabs (Table 1). In the GP/1656/19 group, the virus was detected in the trachea on day 1–11 p.i. and in cloacal swabs on day 3–11 p.i. in the inoculated chickens, the virus can be detected on day 1–11 p.c. (post-contact) in the trachea and on day 3–11 p.c. in cloacal swabs in the contacted chickens (Table 1). In the SW/10429/19 group, the virus can be detected on days 1, 3, 5, 7 in the trachea and on day 5 in cloacal swabs in the inoculated gropp, and viruses can be detected in the trachea on days 3, 5, 7, and in cloaca on days 1 and 3 in the contacted chickens (Table 1). The transmission study indicated that the three H9N2 viruses that belonged to poultry lineage could transmit between chickens without prior adaptation. The GP/1656/19 virus transmitted more efficiently than the CK/932/18 and SW/10429/19 virus. However, the two H9N2 viruses that belonged to wild bird lineage transmitted with low efficiency or did not transmit in chickens (Table 1).

**Table 1.** Transmission of the five H9N2 isolates between chickens

Viruses	Virus shedding/total *^a^*																							
	Inoculated group (d.*p*.i *^b^*)												Contacted group (d.*p*.c *^c^*)											
	Tracheal						Cloacal						Tracheal						Cloacal					
	1	3	5	7	9	11	1	3	5	7	9	11	1	3	5	7	9	11	1	3	5	7	9	11
CK*^d^*/932/18	2/3	2/3	3/3	2/3	0/3	0/3	0/3	0/3	3/3	0/3	0/3	0/3	1/3	1/3	1/3	1/3	1/3	1/3	1/3	1/3	2/3	1/3	1/3	1/3
GP *^e^*/1656/19	3/3	3/3	3/3	3/3	3/3	3/3	0/3	3/3	2/3	1/3	2/3	3/3	3/3	3/3	3/3	3/3	3/3	2/3	0/3	2/3	3/3	1/3	1/3	2/3
WD *^f^*/4870/19	1/3	1/3	0/3	1/3	0/3	0/3	0/3	2/3	1/3	1/3	0/3	0/3	0/3	0/3	1/3	0/3	0/3	1/3	0/3	1/3	1/3	1/3	2/3	2/3
SW*^g^*/10429/19	3/3	3/3	3/3	1/3	0/3	0/3	0/3	0/3	2/3	0/3	0/3	0/3	3/3	3/3	3/3	1/3	0/3	0/3	1/3	1/3	0/3	0/3	0/3	0/3
WD/11452/19	0/3	0/3	0/3	0/3	0/3	0/3	0/3	0/3	0/3	0/3	0/3	0/3	0/3	0/3	0/3	0/3	0/3	0/3	0/3	0/3	0/3	0/3	0/3	0/3

*^a^*Group of 6-week-old SPF chickens (*n *= 3) were inoculated intranasally with 10^6^EID_50_ virus in a volume of 100 µl. The swabs were suspended in 1 ml of PBS and were titrated for virus shedding in eggs.

*^b^*days post-inoculation.

*^c^*days post-contact.

*^d^*chicken

*^e^*green peafowl

*^f^*wild bird

*^g^*swan

We also collected the serum of the inoculated and housed chickens on days 10, 15, and 21, respectively. All the serum of CK/932/18, GP/1656/19, and SW/10429/19 groups was positive to the H9N2 virus according to the HI assay, and the HI antibody titers were at the range from 128 to 2048 (Table S4). Nevertheless, the HI titers of the WD/4870/19 group ranged from 16 to 256, and only two chickens produced HI antibodies in the contact group. Additionally, all the serum of the chickens in the WD/11452/19 group was negative (Table S4). These results suggested that the H9N2 viruses of poultry lineage can induce the chicken to produce the specific antibody quickly in higher titers than the wild bird-originated viruses. Altogether, the poultry lineage H9N2 viruses replicated and transmitted more efficiently in chickens and induced higher HI antibody titer than the wild bird-lineage H9N2 viruses.

### Different replication and virulence of H9N2 AIVs in mice

**Figure 6.**
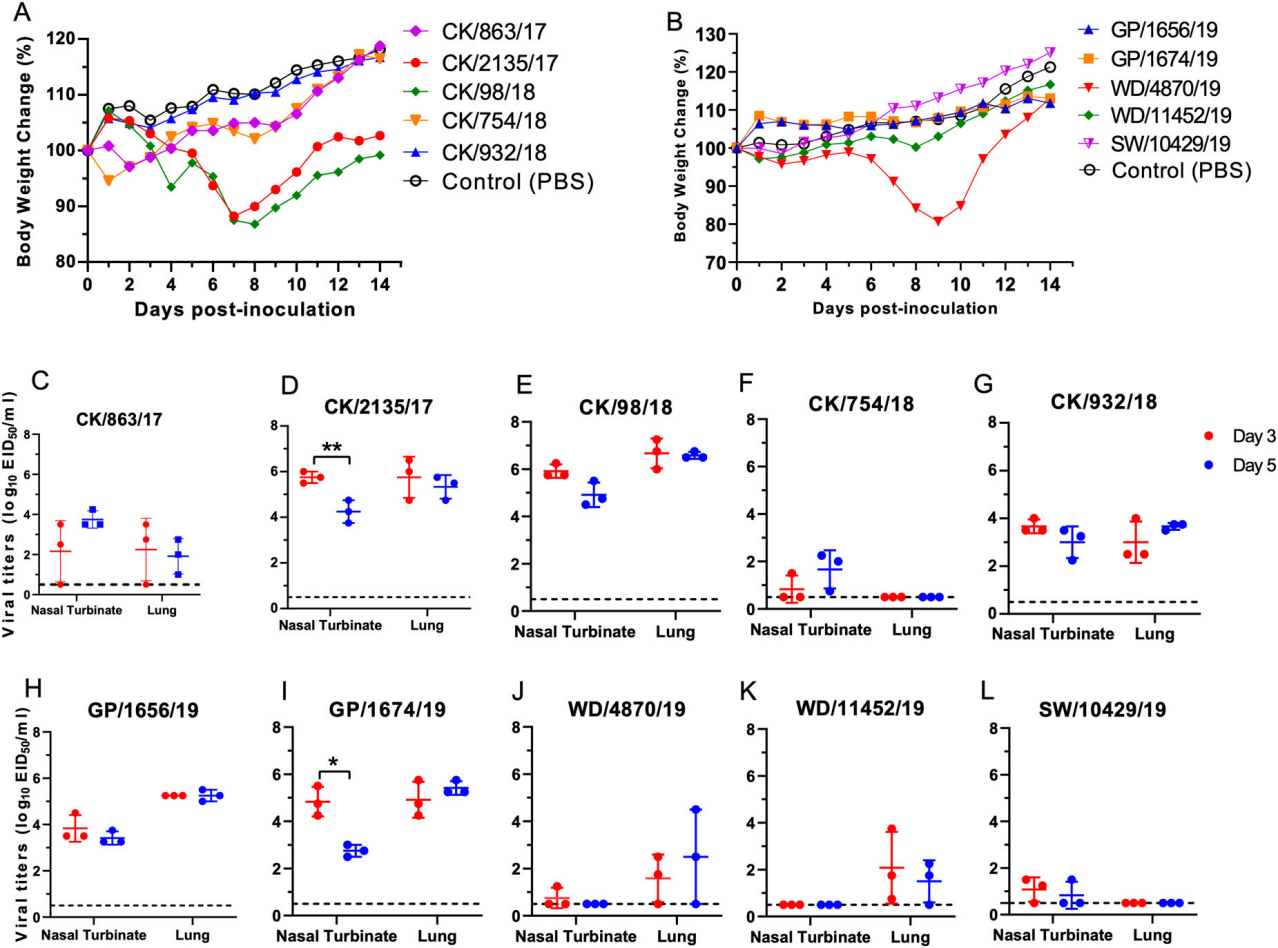
**Virulence and replication of H9N2 viruses in mice.** (A) Bodyweight change of the mice infected with H9N2 viruses isolated from chickens. (B) Bodyweight change of the mice infected with H9N2 viruses isolated from green peafowls and wild birds. Viral titers were detected in eggs (C-L). Data shown are the mean titer from three mice; the error bar indicates the standard deviation. The dashed line indicates the lower limit of detection. Viral titers in the nasal turbinates and lungs of mice at 3 dpi were compared with viral titers at 5 dpi, and significance was tested with a Student's t-test. *, *P* < 0.05; **, *P *< 0.01.

To investigate the potential threat of the H9N2 viruses to mammals, we tested the replication and virulence of ten H9N2 viruses in mice. The selected ten viruses were isolated from different hosts with different isolation times, genotypes, and genetic lineages in the phylogenetic trees. The five chicken H9N2 viruses displayed different replication titers in the nasal turbinate and lung of the mice (Figure 6). The CK/2135/17 and CK/98/18 viruses replicated in the turbinate and lung at higher titers than the other three chicken viruses (Figure 6(c–g)). The viral titer in the lung of the CK/98/18 virus-infected mice was up to 10^6.7^ EID_50_/ml. Additionally, the CK/2135/17 and CK/98/18 viruses led the mice to lose bodyweight up to 11.8% and 13.2%, respectively (Figure 6(a,d,e)). Although the CK/754/18 was clustered into the same genotype with CK/98/18, this virus replicated poorly in the turbinate of the mice, and no viruses were detected in the lungs of the infected mice (Figure 6(f)). The two green peafowl H9N2 viruses shared a similar replication in mice and did not cause body weight loss of the mice during the observation period (Figure 6(b,h,i)). The two wild bird viruses, WD/4870/19 and WD/11452/19, and swan virus SW/10429/19, replicated poorly in the turbinate and lung of the mice. Interestingly, the WD/4870/19 virus can lead to the bodyweight loss of the mice up to 19.4%, although the virus replication titer in turbinate and lung was low (Figure 6(b,j–l)). Hence, the naturally isolated H9N2 viruses from chickens and green peafowl can replicate in the respiratory tract; however, some isolates from chickens, swan, and wild birds still need prior adaptation before they can replicate efficiently in mice. Pathological studies were performed on lung tissues from the infected mice. Most of the lungs of the mice showed mild damage after being infected with the viruses on days 3 and 5, respectively (Figure S4). By contrast, the group's lungs of CK/2135/17 and CK/98/18 showed moderate to severe pulmonary inflammation (Figure S4 B, C, L, M). These pathological changes in the lung of mice in each group were consistent with the replication difference of tested viruses in mice. Summarily, these findings emphasize that the poultry originated H9N2 viruses replicated more efficiently than the wild birds originated viruses, and therefore pose a potential threat to infect human and other mammal animals.

### Replication kinetics and polymerase activity of different H9N2 viruses

**Figure 7.**
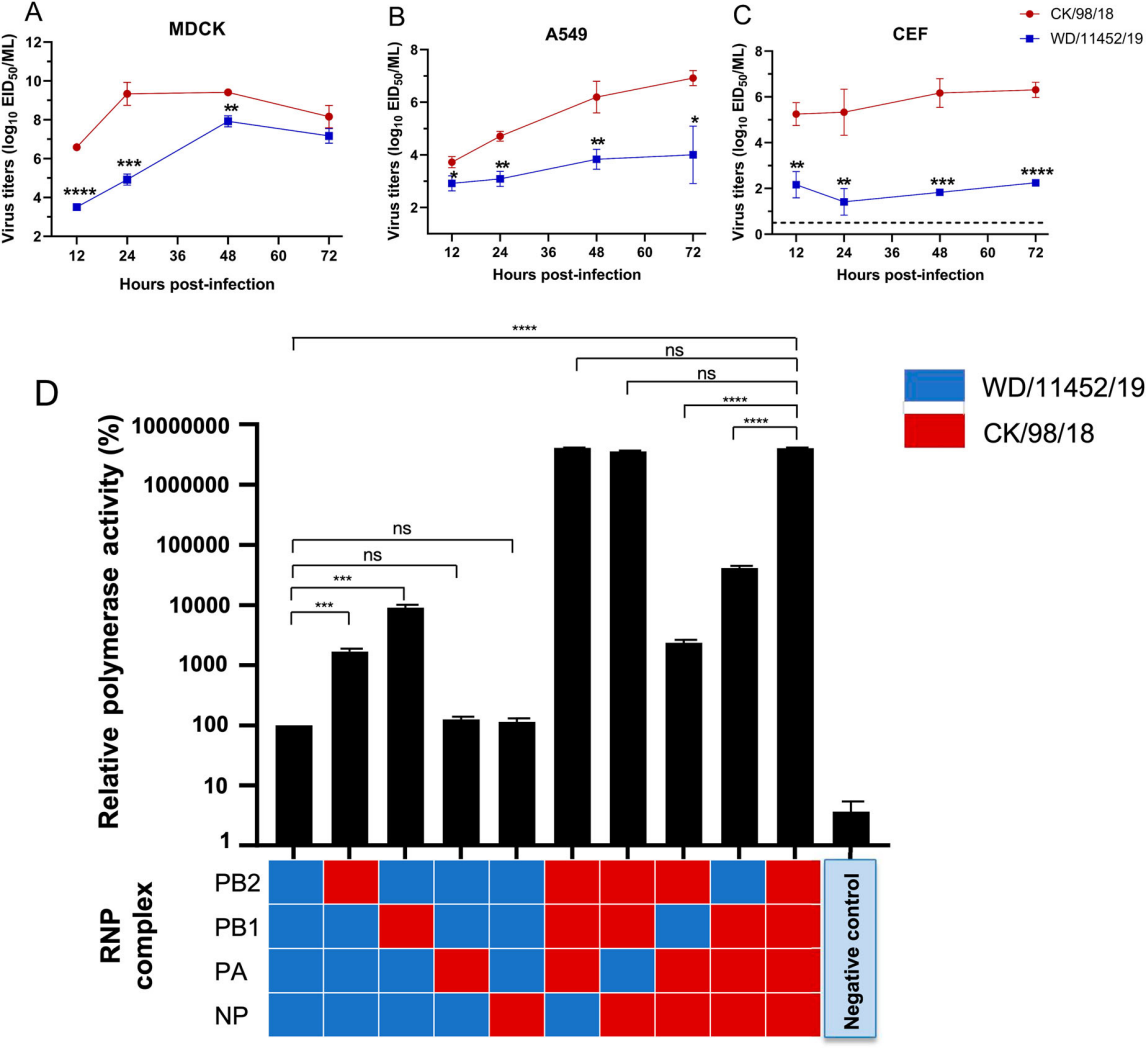
**Virus growth curves and polymerase activity of two H9N2 viruses.** Virus growth curves in MDCK (A), A549 (B), and CEF (C) cells. The dashed lines indicate the lower limit of detection. Data shown are mean ± SD for three independent experiments, and significance was assessed with a two-tailed unpaired Student’s t-test. (D) Polymerase activity of WD/11452/19 and CK/98/18 in 293 T cells. All of the data were normalized to the activity of the WD/11452/19 sample. Data shown are the mean polymerase activity ± standard deviation (n = 3). Statistical analysis was performed by using one-way ANOVA with GraphPad Prism 8 software. *, *P* < 0.05; **, *P* < 0.01; ***, *P* < 0.001; ****, *P* < 0.0001; ns, not significant.

We further investigated the polymerase activity of the poultry lineage virus and the wild bird lineage virus. We selected CK/98/18 and WD/11452/19 as a pair of pattern viruses according to the virus replication in mice. We first performed virus growth curves in mammalian cells (MDCK and A549) and chicken cells (CEF). As shown in Figure 7, the viral titers of CK/98/18 in MDCK, A549, and CEF were significantly higher than the WD/11452/19 viruses. These results were consistent with the virus replication in chickens and mice. Then, we performed a dual-luciferase reporter assay of CK/98/18 and WD/11452/19 in 293 T cells to compare their polymerase activity. The viral polymerase activity of CK/98/18 was higher 30000 times than the WD/11452/19 virus. By single polymerase gene substitution, we found that polymerase genes PB2 and PB1 of CK/98/18 could significantly increase the polymerase activity of WD/11452/19, conversely, the PB2 and PB1 of WD/11452/19 markedly decreased the polymerase activity of CK/98/18 (Figure 7(d)). Together, the differences between the two H9N2 viruses in mammalian cells are consistent with differences in viral polymerase activity.

## Discussion

Here, 16 H9N2 viruses were successfully isolated from chickens, peafowls, swan, and other wild birds, which confirmed that the H9N2 virus could infect various birds. In a previous study, H9N2 viruses have been identified from terrestrial wild birds in North China [16]. These reports indicate that multiple wild bird species are the susceptible hosts of H9N2 influenza viruses. Our surveillance findings emphasize that some H9N2 viruses detected in wild migratory waterfowls and ornamental birds shared the same ancestor with the prevalent poultry viruses. Co-circulation of the two H9N2 lineage viruses in non-poultry, including wild birds, will increase the cross-transmission risk of H9N2 viruses between different avian hosts and certainly add difficulty in disease eradication.

AIVs can be efficiently controlled according to the well-planned and implemented vaccination. For example, vaccination in poultry from 2017 has successfully prevented the prevalence of the H7N9 influenza virus in China and eradicated human infection with the H7N9 virus [46–48]. However, a previous study indicated that up to 57% of the chickens with high HI antibody titer (mean titer was 7.8 log_2_) were infected with the H9N2 virus within five days of being transferred to the LBM [49]. Although the chickens were well vaccinated with H9N2 vaccines, the chickens were not completely protected from H9N2 virus infection in the LBM. The antigenic analysis here shows the poorly cross-reactivity of the chicken serum of poultry H9N2 viruses and indicates that the poultry lineage H9N2 viruses have undergone complicated antigenic drift. Interestingly, the wild bird virus WD/4870/19 can react with both the poultry and wild bird lineage viruses, although we did not find any direct correlation between the HI titer and protein sequence. Thus, a timely, updated, and effective vaccine is urgently needed to prevent the prevalence of the H9N2 virus in poultry.

The receptor-binding property of influenza viruses plays a key role in cross-species transmission [50–53]. Our previous studies have demonstrated that most of the H9N2 influenza viruses isolated in poultry from 2009 to 2013 have acquired the human-like receptor binding specificity, which promoted high transmission of the viruses in ferrets by respiratory droplet [17]. Here, we found that the H9N2 viruses of poultry and wild bird lineage significantly differ in their receptor-binding properties. The H9N2 viruses of wild bird lineage showed similar receptor binding specificity with the poultry lineage viruses prevalent in chickens 20 years ago in China [17]. The E627 K mutation in PB2 has been confirmed to increase the pathogenicity and transmissibility of H9N2 viruses in mammals[54–56]. However, the function of PB2/E627 V mutation in virulence and transmission of H9N2 viruses is still unclear. Thus, continued study is essential to investigate the role of PB2-E627 V mutation in viral phenotype.

The significant difference in molecular characteristics, replication in chickens and mice, transmission in chickens, and polymerase activity between poultry lineage and wild bird lineage viruses imply that the wild bird lineage H9N2 viruses need prior adaptation before replicating and transmitting efficiently in chickens and mammals. Generally, the virulence of influenza viruses is positively correlated with the replication titer in animals. However, the virus WD/4870/19 can lead to severe body weight loss but shows limited replication in mice. The molecular basis responsible for this virulent phenotype still needs to be investigated in our further study.

This study characterized the comparative genetic evolution, receptor-binding specificity, and antigenic diversity and evaluated the replication and transmission of poultry and wild bird lineage H9N2 viruses in chickens and mice. These results further understand the H9N2 viruses prevalent in poultry, wild migratory birds, and other rare birds. Therefore, active control of the prevalence of H9N2 influenza viruses in multiple bird species can decrease the virus's cross-transmission between different avian hosts and reduce human infections with AIVs.

## Supplementary Material

Table_S4.docx

Table_S3.docx

Table_S2.docx

Table_S1.docx

Supplemental_information.docx
