# Supplementary material for: Molecular characterization, receptor binding property, and replication in chickens and mice of H9N2 avian influenza viruses isolated from chickens, peafowls, and wild birds in eastern China
Source: Emerg Microbes Infect. 2021 Nov 12;10(1):2098–112. doi: 10.1080/22221751.2021.1999778 (PMC8592596; doi:10.1080/22221751.2021.1999778)
Supplement: Table_S4.docx [file TEMI_A_1999778_SM1607.docx]

Table S4. Seroconversion of the chickens inoculated or directed contacted with H9N2 influenza viruses

| Viruses | Seroconversion: positive/total *^a^*  (HI antibody titer) | | | | | |
| --- | --- | --- | --- | --- | --- | --- |
|  | Inoculated group (d.p.i *^b^*) | | | Contacted group (d.p.c *^c^*) | | |
|  | 10 | 15 | 21 | 10 | 15 | 21 |
| CK/932/18 | 3/3 (1024, 1024, 512) | 3/3 (1024,1024, 1024) | 3/3 (1024, 512, 1024) | 3/3 (512, 512, 512) | 3/3 (512, 1024, 512) | 3/3 (128, 256, 128) |
| GP/1656/19 | 3/3 (1024,1024, 1024) | 3/3 (512, 256, 512) | 3/3 (2048, 512, 1024) | 3/3 (1024, 512, 2048) | 3/3 (1024, 512, 1024) | 3/3 (2048, 256, 512) |
| WD/4870/19 | 3/3 (64, 32, 32) | 3/3 (128, 64, 64) | 3/3 (256, 256, 256) | 1/3 (64, - *^d^*, -) | 2/3 (64, -, 128) | 1/3 (64, -, 16) |
| SW/10429/19 | 3/3 (1024, 512, 512) | 3/3 (1024, 512, 256) | 3/3 (512, 216, 512) | 3/3 (1024, 1024, 512) | 3/3 (512, 512, 512) | 3/3 (256, 128, 256) |
| WD/11452/19 | 0/3 (-, -, -) | 0/3 (-, -, -) | 0/3 (-, -, -) | 0/3 (-, -, -) | 0/3 (-, -, -) | 0/3 (-, -, -) |

*^a^* The chicken serum was collected on days 10, 15, and 21 p.i. The HI antibody titers were detected by HI assay. The five H9N2 viruses were diluted to 4 HAU and then reacted with the chicken serum, e.g., the CK/932/18 was diluted to 4 HAU and then reacted with the chicken serum of the CK/932/18 group.

*^b^* days post-inoculation.

*^c^* days post-contact.

*^d^* The HI antibody titer was negative.
