# Supplementary material for: Molecular characterization, receptor binding property, and replication in chickens and mice of H9N2 avian influenza viruses isolated from chickens, peafowls, and wild birds in eastern China
Source: Emerg Microbes Infect. 2021 Nov 12;10(1):2098–112. doi: 10.1080/22221751.2021.1999778 (PMC8592596; doi:10.1080/22221751.2021.1999778)
Supplement: Table_S3.docx [file TEMI_A_1999778_SM1606.docx]

Table S3. Cross-reactive hemagglutinin inhibition (HI) antibody titers of the sixteen H9N2 viruses with different chicken antisera.

| Viruses | HI antibody titer of chicken antiserum *^a^* | | | | |
| --- | --- | --- | --- | --- | --- |
|  | CK/932/18 | GF/1656/19 | SW/10429/19 | WD/11452/19 | WD/4870/19 |
| CK/863/17 | 256 | 128 | 128 | 32 | 64 |
| CK/2104/17 | 4 | 32 | 2 | 2 | 8 |
| CK/2135/17 | 128 | 512 | 16 | 64 | 64 |
| CK/754/17 | 2 | 64 | / *^b^* | / | 4 |
| CK/98/18 | 128 | 512 | 8 | 32 | 32 |
| CK/932/18 | **256 *^c^*** | 512 | 32 | 64 | 64 |
| GF/1656/19 | 64 | **1024** | 8 | 16 | 32 |
| GF/1674/19 | 64 | 512 | 8 | 32 | 32 |
| SW/10429/19 | 128 | 256 | **512** | 128 | 64 |
| WB/11706/19 | 8 | 16 | 2 | 16 | 64 |
| WD/11449/19 | 64 | 256 | 64 | 64 | 512 |
| WD/11452/19 | 32 | 128 | 32 | **64** | 256 |
| WD/11442/19 | 8 | 32 | 2 | 32 | 128 |
| WB/11187/19 | 16 | 64 | 4 | 64 | 64 |
| WD/4629/19 | 8 | 16 | 2 | 16 | 128 |
| WD/4870/19 | 32 | 64 | 8 | 64 | **512** |

1. The antisera were generated by infecting SPF chickens with the H9N2 viruses, as indicated in this table.
2. The cross-reactive HI antibody titer was 0.
3. The homologous titer is shown in bold.
