# Supplementary material for: Molecular characterization, receptor binding property, and replication in chickens and mice of H9N2 avian influenza viruses isolated from chickens, peafowls, and wild birds in eastern China
Source: Emerg Microbes Infect. 2021 Nov 12;10(1):2098–112. doi: 10.1080/22221751.2021.1999778 (PMC8592596; doi:10.1080/22221751.2021.1999778)
Supplement: Table_S2.docx [file TEMI_A_1999778_SM1605.docx]

Table S2. Amino acid substitutions of the H9N2 viruses contribute to enhanced replication, pathogenicity, and transmissibility in mammals.

| Viruses | HA (H3 Numbering) | | | | | NA | PB2 | | | | PB1 | | | PA | M1 | | NS1 |
| --- | --- | --- | --- | --- | --- | --- | --- | --- | --- | --- | --- | --- | --- | --- | --- | --- | --- |
|  | I155T | H183N | A190V | Q226L | Cleavage site | Amino acids deletion at position 63-65 | T271A | Q591K | E627K | D701N | R207K | I368V | H436Y | A515T | N30D | T215A | P42S |
| CK /2135/17 | T | N | A | L | KSSR.GLF | Yes | T | Q | V | D | K | V | Y | T | D | A | S |
| CK /98/18 | T | N | V | L | RSSR.GLF | Yes | T | Q | V | D | K | V | Y | T | D | A | S |
| CK/932/18 | T | N | A | L | RSRR.GLF | Yes | T | Q | V | D | K | V | Y | T | D | A | S |
| GP/1656/19 | T | N | V | L | RSSR.GLF | Yes | T | Q | E | D | K | V | Y | T | D | A | S |
| GP/1674/19 | T | N | A | L | RSSR.GLF | Yes | T | Q | E | D | K | V | Y | T | D | A | S |
| CK/863/17 | T | N | T | L | RSSR.GLF | Yes | T | Q | E | D | K | V | Y | T | D | A | S |
| CK/2104/17 | T | N | A | L | RSSR.GLF | Yes | T | Q | V | D | K | V | Y | T | D | A | S |
| CK/754/18 | T | N | T | L | RSSR.GLF | Yes | T | Q | E | D | K | V | Y | T | D | A | S |
| SW/10429/19 | T | N | T | L | RSSR.GLF | Yes | T | Q | E | D | K | V | Y | T | D | A | S |
| WB/4629/19 | T | H | E | Q | ASNR.GLF | No | T | Q | E | D | K | M | Y | T | D | A | S |
| WB/4870/19 | T | H | E | Q | ASNR.GLF | No | T | Q | E | D | K | M | Y | T | D | A | S |
| WD/11706/19 | T | H | E | Q | ASNR.GLF | No | T | Q | E | D | K | I | Y | T | D | A | A |
| WB/11449/19 | T | H | E | Q | ASNR.GLF | No | T | Q | E | D | K | I | Y | T | D | A | S |
| WB/11452/19 | T | H | E | Q | ASNR.GLF | No | T | Q | E | D | K | I | Y | T | D | A | S |
| WB/11442/19 | T | H | E | Q | ASNR.GLF | No | T | Q | E | D | K | I | Y | T | D | A | S |
| WB/11187/19 | T | H | E | Q | ASNR.GLF | No | T | Q | E | D | K | I | Y | T | D | A | S |
