# Supplementary material for: Molecular characterization, receptor binding property, and replication in chickens and mice of H9N2 avian influenza viruses isolated from chickens, peafowls, and wild birds in eastern China
Source: Emerg Microbes Infect. 2021 Nov 12;10(1):2098–112. doi: 10.1080/22221751.2021.1999778 (PMC8592596; doi:10.1080/22221751.2021.1999778)
Supplement: Table_S1.docx [file TEMI_A_1999778_SM1604.docx]

Table S1. Sixteen H9N2 viruses were isolated from chickens, green peafowls, and wild birds in eastern China from 2017 to 2019.

| Viruses | Abbreviation | Accession numbers | Isolation locations | Collection date | Host | Specimen source | Sample numbers | Identified subtypes |
| --- | --- | --- | --- | --- | --- | --- | --- | --- |
| A/chicken/Shandong/863/2017(H9N2) | CK/863/17 | MZ502907-MZ502914 | Chicken farm,  Shandong, China | May 10, 2017 | Chicken | Throat and cloacal swabs | 15 | H9N2(4) |
| A/chicken/Shandong/2104/2017(H9N2) | CK/2104/17 | MZ502915-  MZ502922 | Live poultry market, Shandong, China | Dec 6, 2017 | Chicken | Throat and cloacal swabs | 25 | H9N2(1) |
| A/chicken/Shandong/2135/2017(H9N2) | CK/2135/17 | MW389307- MW389314 | Live poultry market, Shandong, China | Nov 26, 2017 | Chicken | Throat and cloacal swabs | 20 | H3N2(2)  H9N2(4) |
| A/chicken/Shandong/98/2018(H9N2) | CK/98/18 | MW389299- MW389306 | Live poultry market, Shandong, China | Jan 23, 2018 | Chicken | Throat and cloacal swabs | 40 | H9N2(3) |
| A/chicken/Shandong/754/2018(H9N2) | CK/754/18 | MZ502931-MZ502938 | Live poultry market, Shandong, China | Apr 22, 2018 | Chicken | Throat and cloacal swabs | 13 | H9N2(1) |
| A/chicken/Shandong/932/2018 (H9N2) | CK/932/18 | MW389291- MW389298 | Live poultry market, Shandong, China | Apr 29, 2018 | Chicken | Throat and cloacal swabs | 17 | H9N2(1) |
| A/green peafowl/Shandong/1656/2019(H9N2) | GP/1656/19 | MW389275- MW389282 | Wildlife park, Shandong, China | Feb 22, 2019 | Green peafowl | Fresh droppings | 73 | H9N2(2) |
| A/green peafowl/Shandong/1674/2019(H9N2) | GP/1674/19 | MW389283- MW389290 |  |  |  |  |  |  |
| A/wild bird /Shandong/4629/2019(H9N2) | WB/4629/19 | MZ502947-MZ502954 | Waterfowl habitats, Yellow River Delta, Shandong, China | Sep 27, 2019 | Migratory waterfowl | Fresh droppings | 848 | H4(2)  H4N6(1)  H6(1)  H6N6(1) H9(2)  H9N2(2) |
| A/wild bird /Shandong/4870/2019(H9N2) | WB/4870/19 | MZ502899-MZ502906 |  |  |  |  |  |  |
| A/swan/Shandong/10429/2019(H9N2) | SW/10429/19 | MZ502883-MZ502890 | Wetland, Shandong, China | Nov 20, 2019 | Swan | Fresh droppings | 574 | H9(1)  H9N2(1) |
| A/wild bird /Shandong/11187/2019(H9N2) | WB/11187/19 | MZ502891-MZ502898 | Waterfowl habitats, Yellow River Delta, Shandong, China | Dec 15, 2019 | Migratory waterfowl | Fresh droppings | 1191 | H3(2)  H3N1(1)  H3N8(1)  H4N2(1)  H4N6(2)  H6N1(1)  H9(3)  H9N2(5) |
| A/wild bird /Shandong/11442/2019(H9N2) | WB/11442/19 | MZ502923-MZ502930 |  |  |  |  |  |  |
| A/wild bird /Shandong/11449/2019(H9N2) | WB/11449/19 | MZ502875-MZ502882 |  |  |  |  |  |  |
| A/wild bird /Shandong/11452/2019(H9N2) | WB/11452/19 | MZ502939-MZ502946 |  |  |  |  |  |  |
| A/wild bird /Shandong/11706/2019(H9N2) | SW/11706/19 | MZ502867-MZ502874 |  |  |  |  |  |  |
