# Supplementary material for: Molecular characterization, receptor binding property, and replication in chickens and mice of H9N2 avian influenza viruses isolated from chickens, peafowls, and wild birds in eastern China
Source: Emerg Microbes Infect. 2021 Nov 12;10(1):2098–112. doi: 10.1080/22221751.2021.1999778 (PMC8592596; doi:10.1080/22221751.2021.1999778)
Supplement: Supplemental_information.docx [file TEMI_A_1999778_SM1603.docx]

Supplemental information


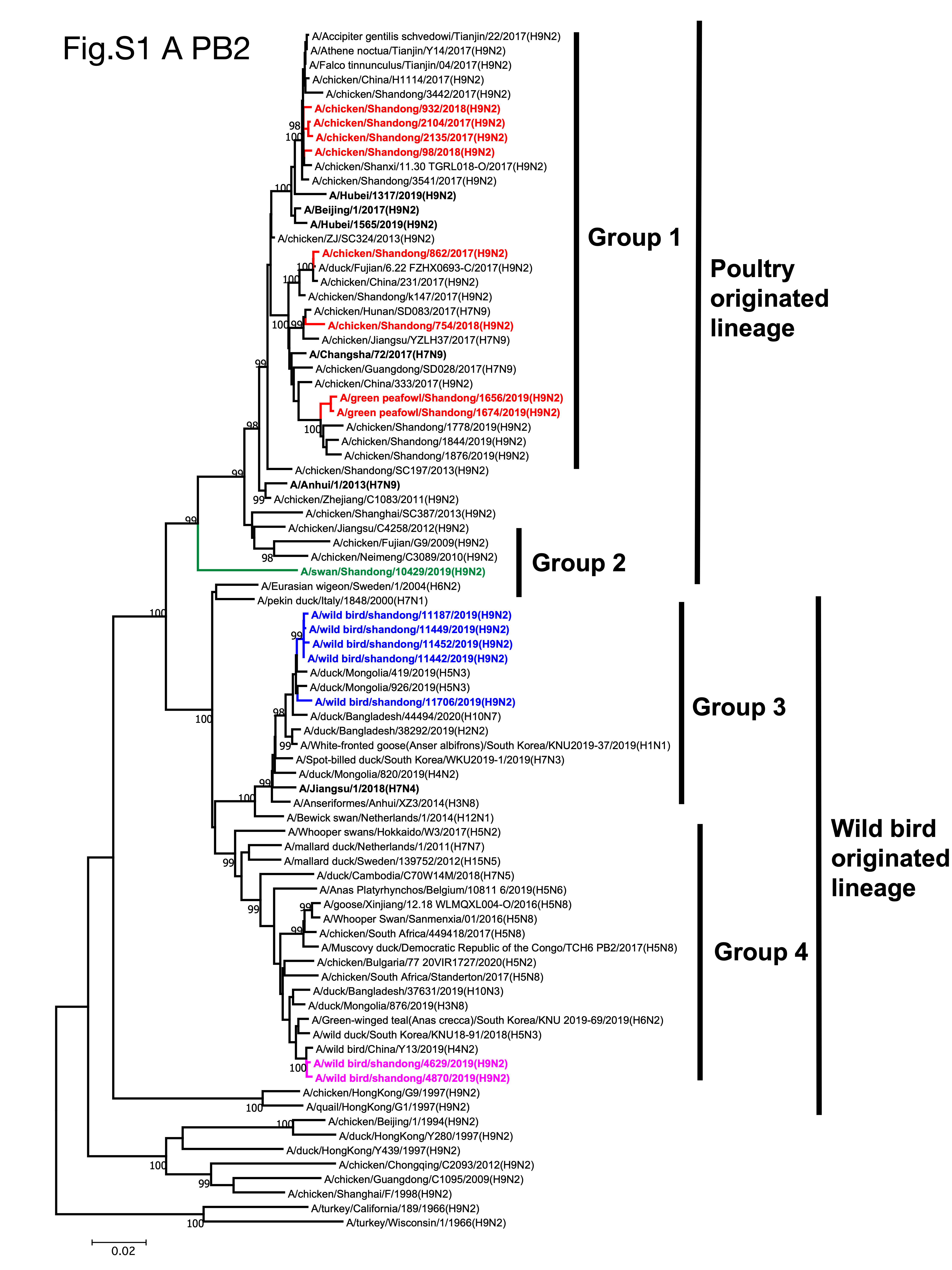


**
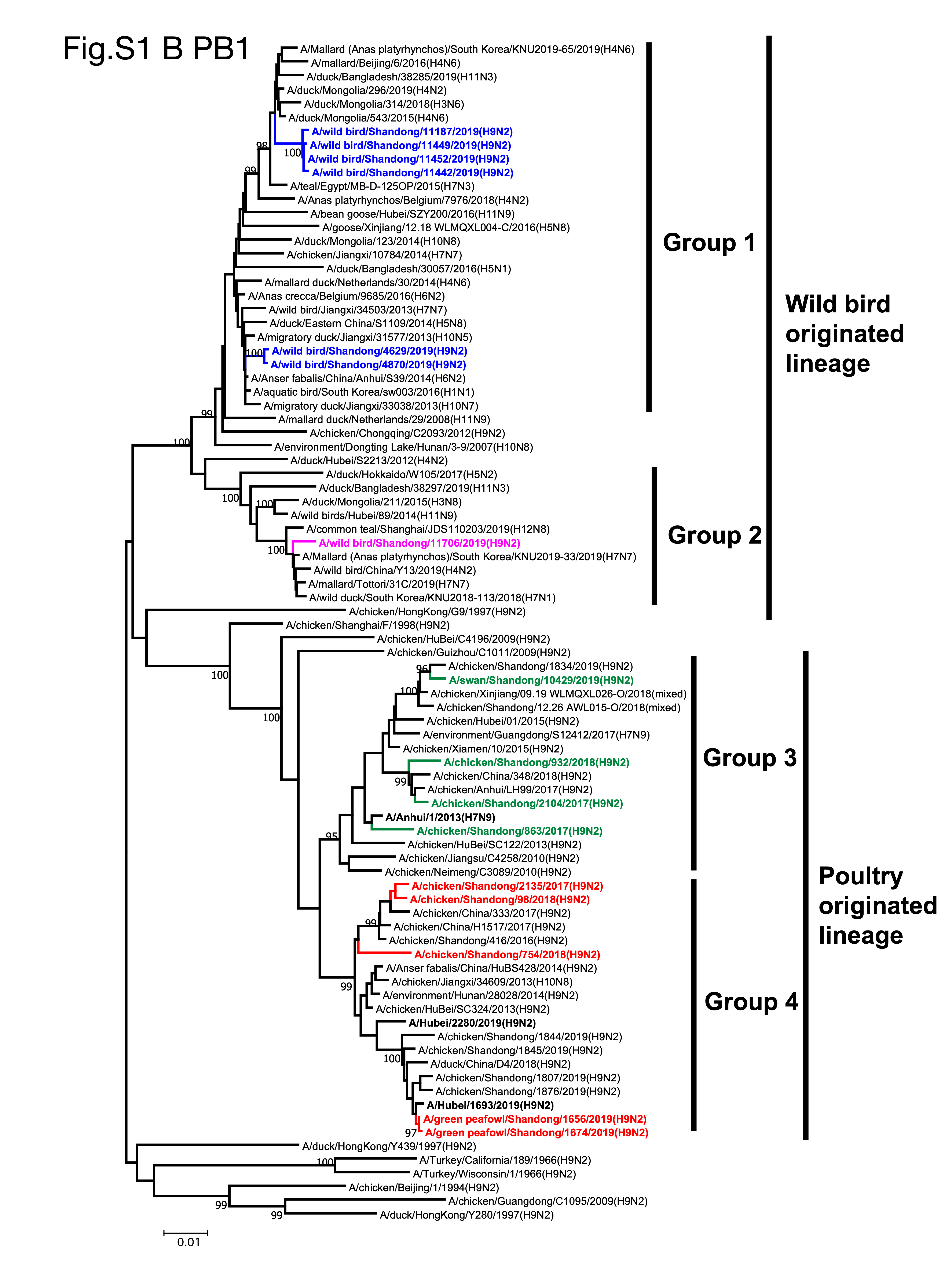
**

**
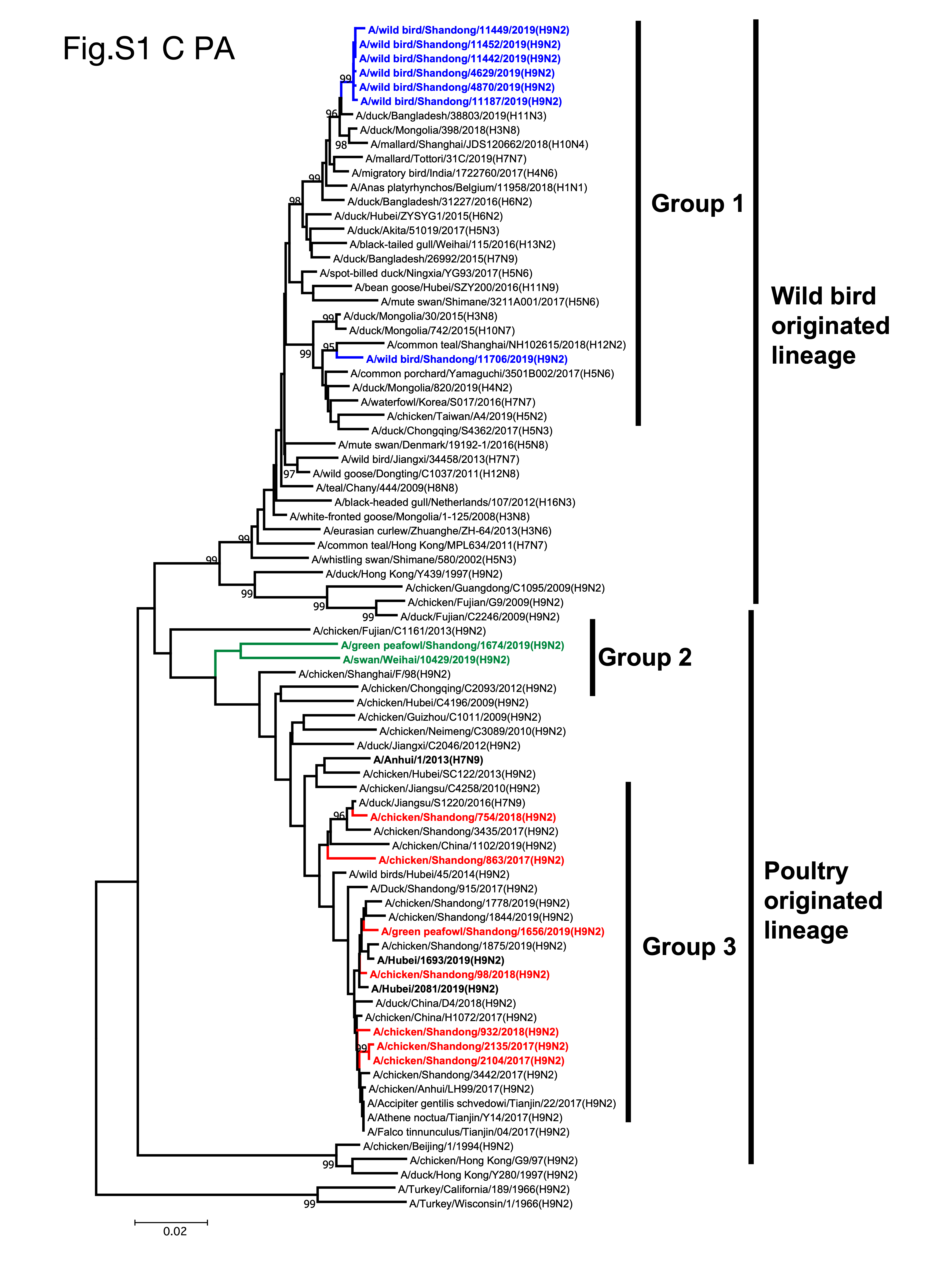
**

**
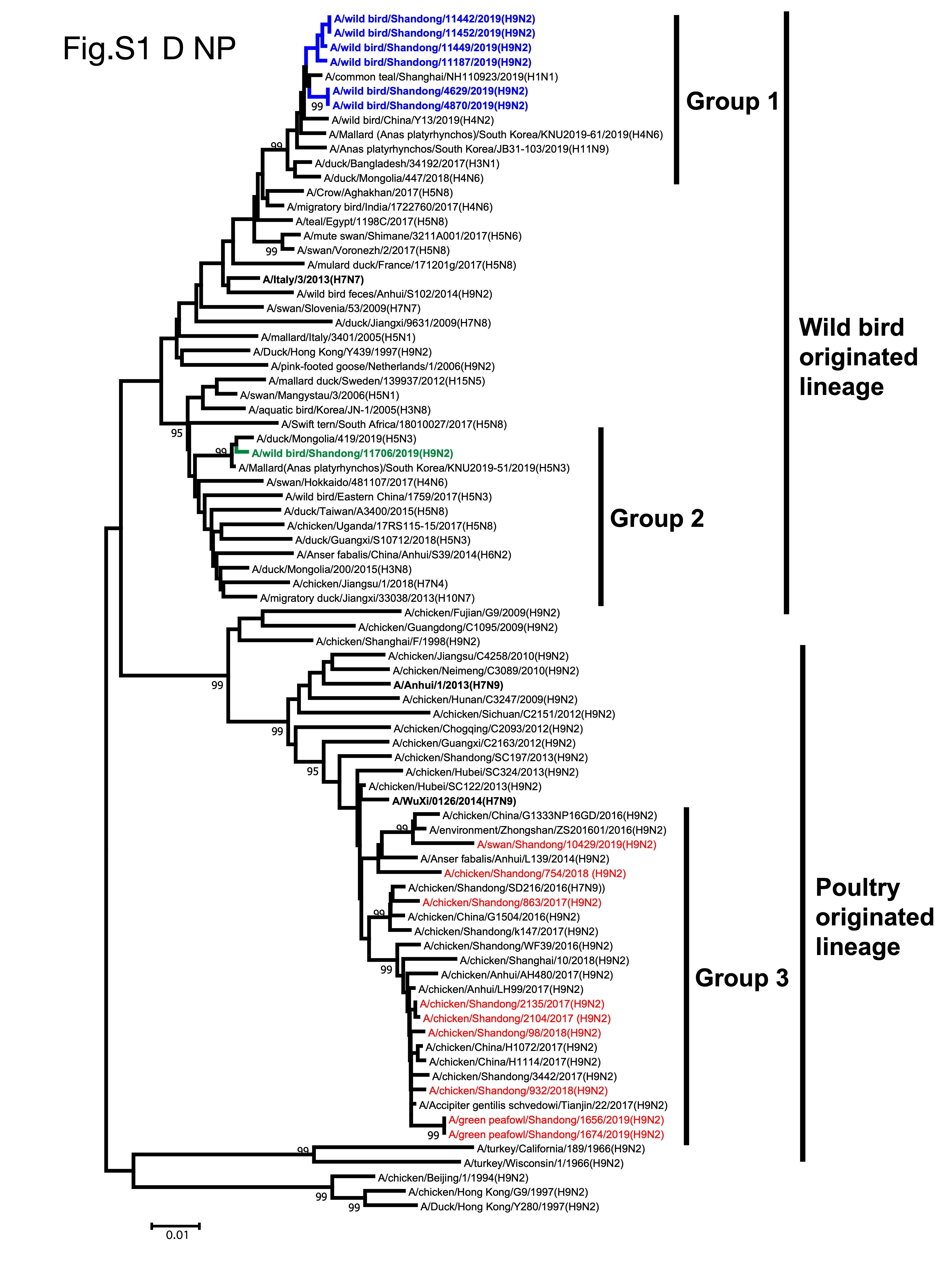
**

**
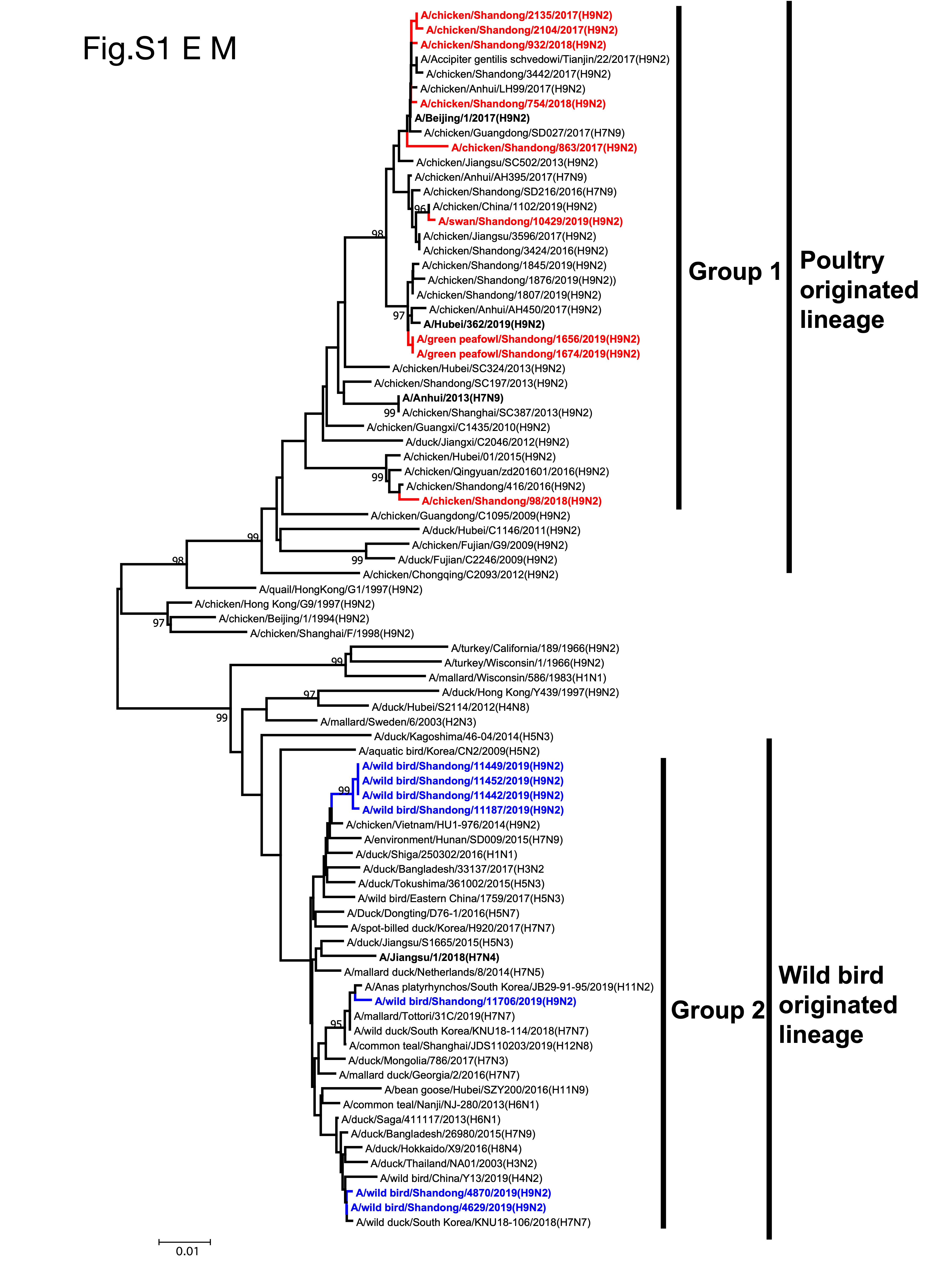
**

**
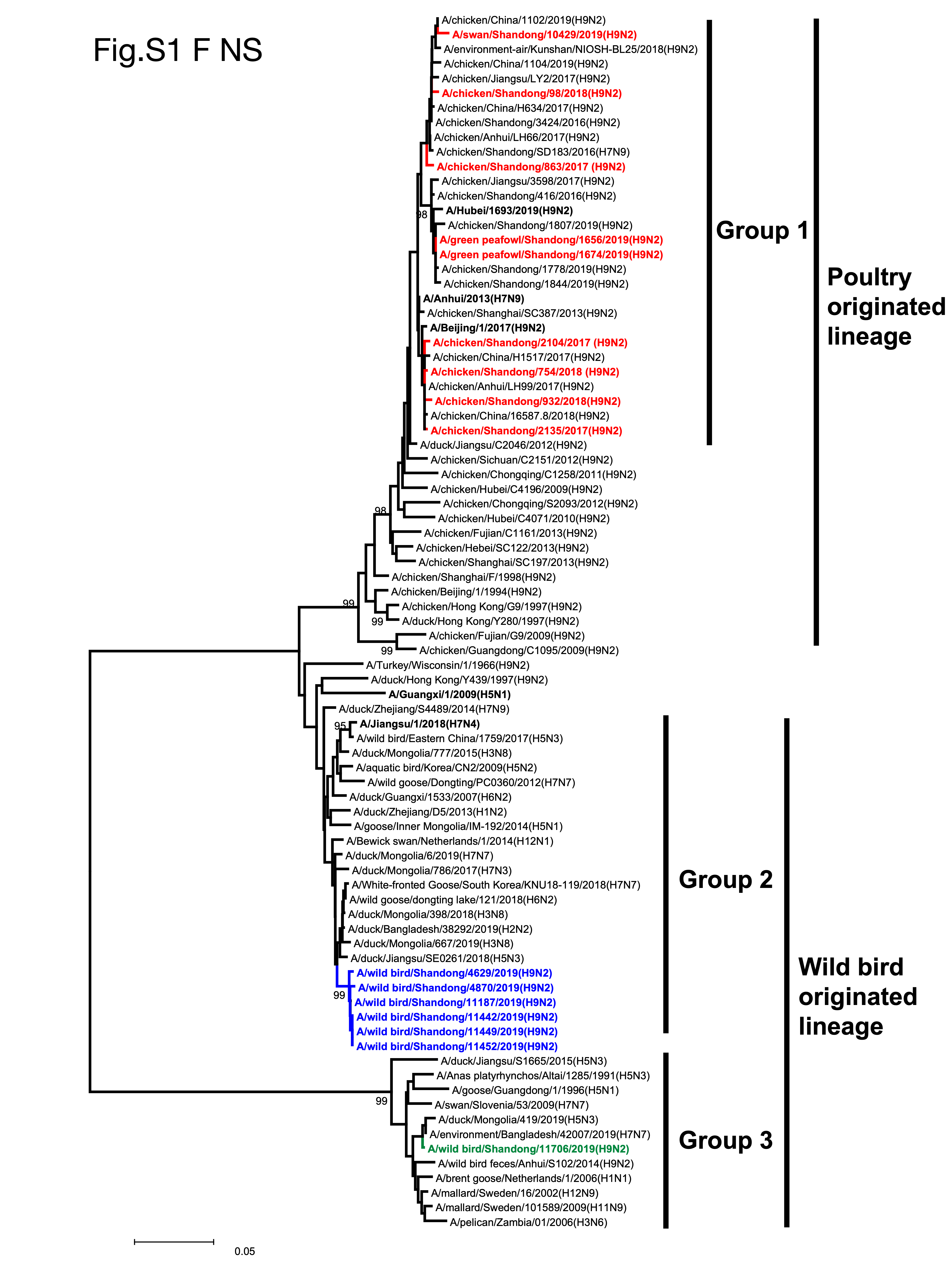
**

**Figure S1. Phylogenetic trees of the internal genes of H9N2 viruses.** PB2 (A), PB1 (B), PA (C), NP (D), M (E), NS (F).

**
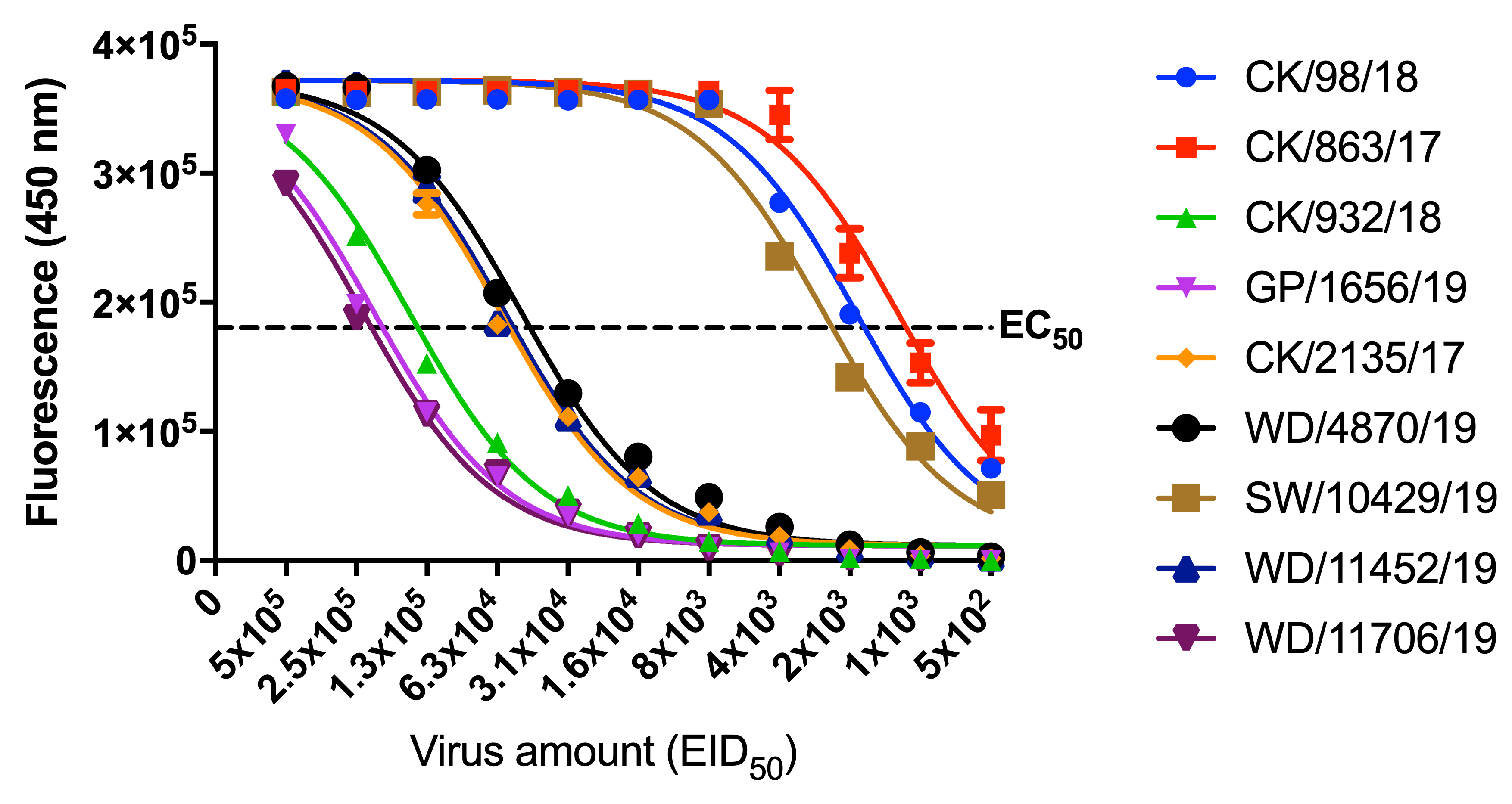
**

**Figure S2. Neuraminidase activities of nine H9N2 viruses.** The neuraminidase activity was analyzed in the presence of the substrate MUNANA. The reaction was analyzed at excitation and emission wavelengths of 365 nm and 450 nm, respectively, and performed in triplicate. The dashed line shows the half-maximal effective concentration (EC_50_).


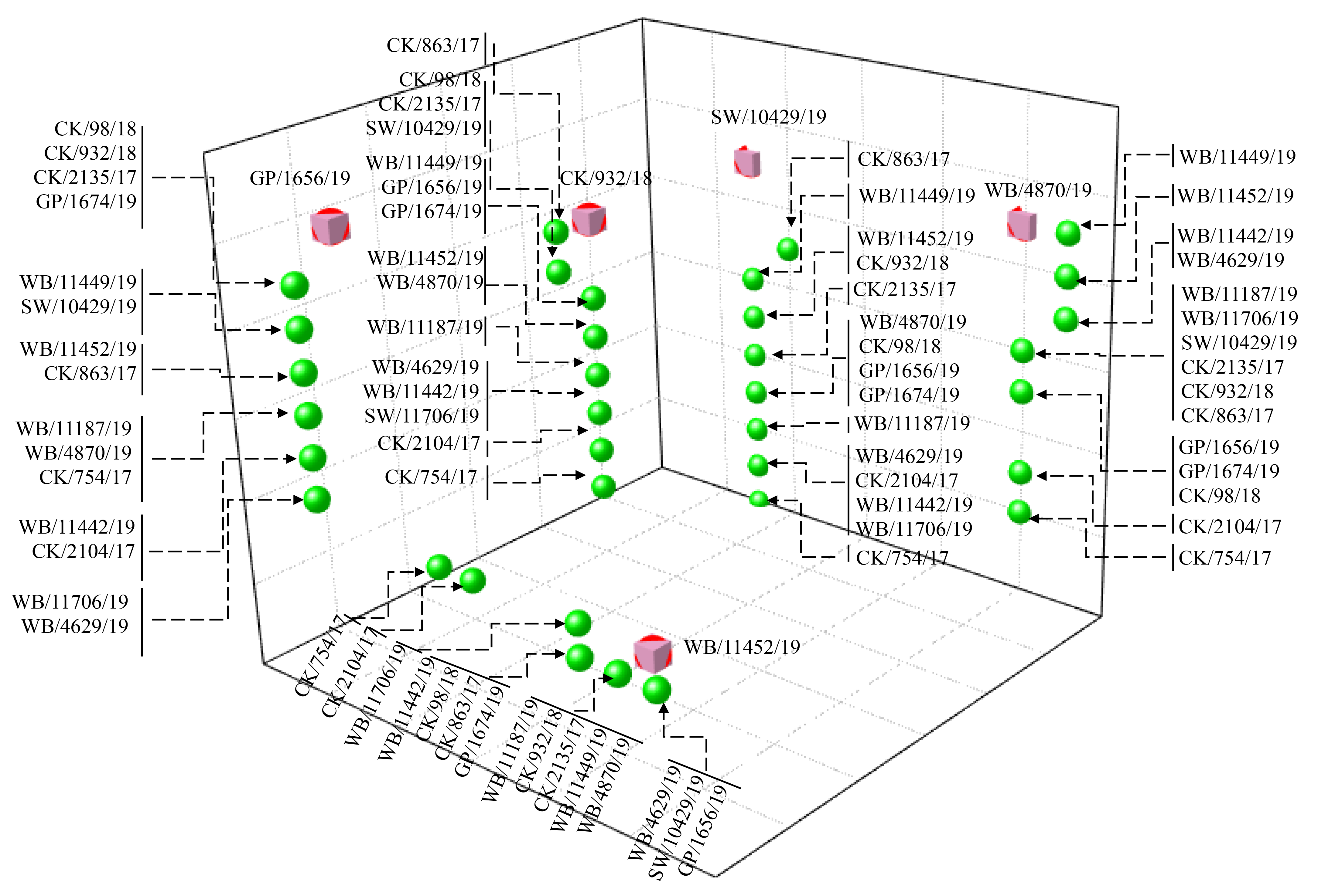


**Figure S3. Antigenic map of the H9N2 viruses.** Antigenic analysis was performed by using the hemagglutinin inhibition (HI) assay with 1% chicken erythrocyte. The antigenic map of the H9N2 viruses used in this study was generated using the HI assay data in Origin software.


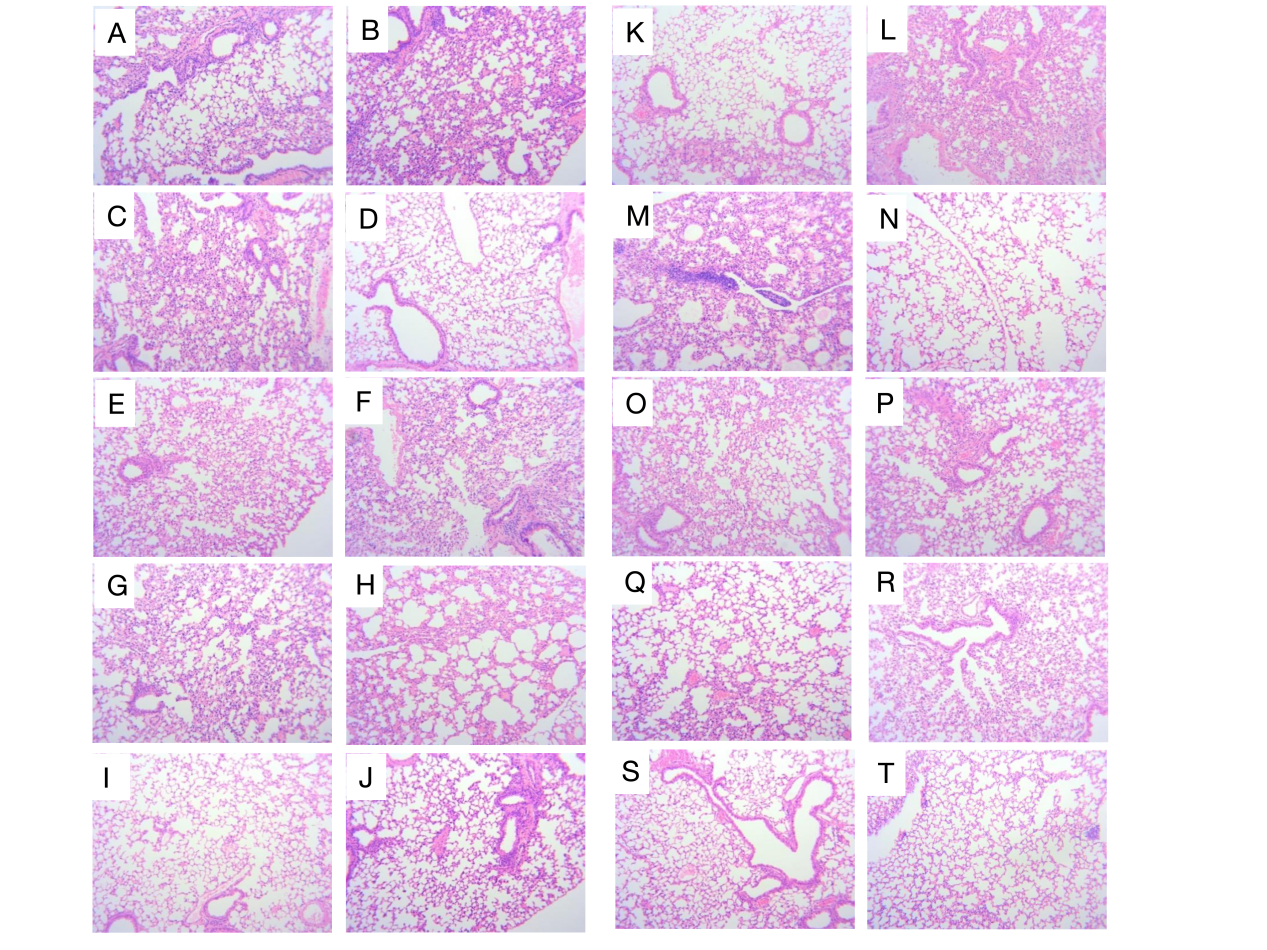


**Figure S4. Pathological study of the lung samples of the mice infected with H9N2 viruses.** Lungs were sampled for histologic study. The lung samples were collected, fixed in 10% formalin, and then stained with hematoxylin and eosin (HE). (A) CK/863/17; (B) CK/2135/17; (C) CK/98/18; (D) CK/754/18; (E) CK/932/18; (F) GP/1656/19; (G) GP/1674/19; (H) WD/4870/19; (I) WD/11452/19; (J) SW/10429/19, the lung pathological changes of the mice infected with the viruses at day 3 p.i.. (K) CK/863/17; (L) CK/2135/17; (M) CK/98/18; (N) CK/754/18; (O) CK/932/18; (P) GP/1656/19; (Q) GP/1674/19; (R) WD/4870/19; (S) WD/11452/19; (T)SW/10429/19, the pathological lung changes of the mice infected with the viruses at day 5 p.i.
